# Screening for High-Yielding Pyruvate and Acetaldehyde Yeasts and Their Application in Improving the Stability of Anthocyanin in Mulberry Wine

**DOI:** 10.3390/foods14132278

**Published:** 2025-06-27

**Authors:** Hui Zhou, Yajie Chai, Weidong Huang, Jicheng Zhan, Yilin You

**Affiliations:** 1 Yantai Institute of China Agricultural University, Binhai Middle Road 2006, Laishan District, Yantai 264670, China; huizhou200916@163.com (H.Z.); weidonghuang@cau.edu.cn (W.H.); 2 College of Food Science and Nutritional Engineering, Beijing Key Laboratory of Viticulture and Enology, China Agricultural University, Tsinghua East Road 17, Haidian District, Beijing 100083, China

**Keywords:** wild wineyeast, color stability, pyruvate acid, acetaldehyde, mulberry wine

## Abstract

The structure of anthocyanins in mulberry wine is highly unstable and, therefore, degrades during the fermentation and aging process. This instability hinders the maintenance of color, affects the sensory quality, and impedes the development of the mulberry wine industry. In this study, high-yielding acetaldehyde yeasts *Saccharomyces cerevisiae* GS32 and *Candida glabrata* GS13, as well as high-yielding pyruvate yeast *Candida xestobii* D1, were selected from our laboratory’s strain bank for application in mulberry wine fermentation. The objective was to determine the impact of these high-yielding yeasts on improving anthocyanin content and color stability. The results revealed that different strains and inoculation methods significantly influenced anthocyanin content and color stability in mulberry wine. The GS32 exhibited the highest increase in total anthocyanin content, while the D1 showed a superior retention rate for C3G (a specific type of anthocyanin). Over a period of 1–5 weeks of aging time, minimal changes in color were observed across all treatment groups. These findings suggest that fermenting with yeast strains that yield high levels of pyruvate and acetaldehyde is an effective approach to address issues related to the poor stability of anthocyanins in mulberry wine.

## 1. Introduction

Mulberry is a fruit that is used both as food and medicine. It contains 18 kinds of amino acids, vitamin C, selenium, and other trace elements, as well as macro elements such as potassium and magnesium [1,2]. The research shows that mulberry has anti-oxidation, lowers blood sugar, and enhances immunity effects [3,4]. At present, mulberry trees are widely distributed, with a rich variety of species and considerable output. Over the years, they have played a positive role in the process of world economic development. However, the direct selling of mulberries is hampered by its fragile and thin skin. To solve this problem, its processing is needed to extend its shelf life. Therefore, mulberry is marketed as a juice, wine, and vinegar.

As the highest value-added product derived from mulberry processing, this mulberry wine is the highest-valued market. This beverage not only effectively harnesses the nutritional and bioactive components of mulberries but also addresses challenges related to their seasonality and short shelf life. However, during fermentation and aging, mulberry wine encounters a critical issue of color degradation, which substantially hampers its marketability. The primary reason for this phenomenon is the poor stability of anthocyanins, the main coloring agents in mulberry wine. Due to their structural characteristics—featuring multiple reactive hydroxyl groups and a positively charged core—anthocyanins exhibit instability, rendering them susceptible to external factors such as pH levels and temperature fluctuations, as well as interactions with metal ions like calcium and copper, along with enzymatic activities involving glycoside hydrolases and polyphenol oxidases [5,6,7].

There are several methods to enhance the stability of anthocyanins: lowering the pH of the wine, improving or optimizing production processes to reduce the loss of anthocyanins during production, and utilizing auxiliary pigments to form anthocyanin–auxiliary pigment complexes to increase the stability of anthocyanins [8,9,10,11,12,13]. Previous studies on improving the stability of anthocyanins mainly utilized the auxochrome effect. However, in our previous study on protecting the color of mulberry wine using auxiliary pigments (yeast extract and tannins), the results showed that simply utilizing the auxochrome effect was not suitable for mulberry wine aging for more than four months [14,15]. Therefore, it is necessary to seek other methods that can improve the stability of anthocyanins in mulberry wine and slow down the fading of the wine.

Yeast strains are increasingly being recognized as a potential biological tool by researchers. It has been discovered that yeast can enhance the formation of stable pigments by producing and releasing metabolites. From the perspective of promoting the formation of VPA (volatile phenylacetates), this research group has screened strains with high HCDC (hydroxycinnamic acid decarboxylase) yield and applied them to investigate color protection in mulberry wine. The results showed that these strains effectively address the issue of color instability in mulberry wine [16]. Furthermore, specific yeast metabolites such as pyruvate and acetaldehyde have distinct impacts on the formation of vitisin A and vitisin B (anthocyanins found primarily in red wines), respectively. Currently, the utilization of yeast strains with elevated levels of pyruvate and acetaldehyde to improve anthocyanin stability is primarily focused on wine production [16]. Therefore, further investigation is warranted to determine if similar effects can be observed in mulberry wine.

The goal of this research is to incorporate novel yeast to preserve the quality of mulberry wine, thereby lengthening its shelf life and increasing its market size in China. In our research group, a total of 501 wild yeast strains were isolated from eight major wine-producing regions in China. These strains were combined with the literature reports on high-yielding pyruvate and acetaldehyde yeast strains. For this experiment, 78 strains of *Saccharomyces cerevisiae* and 32 strains of non-*Saccharomyces cerevisiae* were screened for the production of pyruvic acid and acetaldehyde for application to mulberry wine fermentation. We determined the basic physical and chemical indexes as well as color parameters of mulberry wine produced by these selected yeast strains.

## 2. Materials and Methods

### 2.1. Mulberry, Yeasts, and Reagents

Mulberry was black mulberry purchased from Shantou, Guangdong. Seventy-eight strains of *Saccharomyces cerevisiae* and thirty-two strains of *non-Saccharomyces cerevisiae* isolated from vineyards and wine in Beijing, Ningxia, Gansu, Xinjiang, etc., and preserved in our laboratory were included in the screening for high-yield of pyruvate and acetaldehyde. The specific information is shown in Table S1. Commercial saccharomyces Intertius AMR-1 was selected as the control yeast strain. Formic acid and acetonitrile (HPLC grade; ≥99.9%) were purchased from Fisher (Waltham, MA, USA). Glucose, fructose, glycerol, gallic acid, and centaurin-3-O-glucoside standard substances (HPLC grade; ≥99.9%) were purchased from Sigma-Aldrich (St. Louis, CA, USA). Peptone, agar powder, all analytical reagents, and other commonly used reagents were purchased from Sinopharm Chemical Reagent Co., Ltd. (Shanghai, China).

### 2.2. Primary Screening of Yeast with High Yields of Pyruvate and Acetaldehyde

The purified single colony was inoculated in a YPD liquid medium and cultured at 28 °C and 180 r/min for 24 h to obtain a single yeast solution. By adjusting the OD_600_ value of the single bacterial solution to 1.1 with 0.85% normal saline, yeast pre-culture solution was obtained. Then, the pre-culture solution was inoculated in a YPD liquid medium and cultured at 25 °C for 5 d. The contents of acetaldehyde and pyruvate in the supernatant diluted 4 times were determined.

#### 2.2.1. The Mehod of Detection of Acetaldehyde

Acetaldehyde content was determined according to Romano et al. [17] with slight modification using high-performance liquid chromatography (HPLC). The method was as follows:

Sample treatment: 0.5 mL of supernatant of fermentation broth of the test yeast strain was taken, 200 μL of 4 mol/L NaOH solution was added, mixed and left to stand for 10 min, then 0.5 mL of acetonitrile, 150 μL of 25% sulphuric acid solution, and 200 μL of 10 g/L of 2,4-dinitrophenylhydrazine solution were added, mixed and left to stand for 15 min.

Chromatographic conditions: chromatographic column: xBridge^®^C18 (4.6 mm × 150 mm, 5 μm); column temperature: 30 °C; wavelength: 360 nm; mobile phase: acetonitrile/water = 65:35; flow rate: 0.5 mL/min; injection volume: 10 μL; the temperature of sample chamber: 4 °C.

#### 2.2.2. The Mehod of Detection of Pyruvate

Pyruvate was measured according to Wolff et al. [18] with slight modifications. The method was as follows:

An amount of 1 mL of the supernatant of the fermentation broth of the test yeast strain was taken, 2 mL of 8% trifluoroacetic acid, 1 mL of 0.1% 2,4-dinitrophenylhydrazine solution, and 5 mL of 1.5 mol/L NaOH solution was added, mixed well, and left to stand for 15 min to determine the absorbance at 520 nm.

### 2.3. Secondary Screening for Yeast Strains with Strong Stress Resistance and Excellent Fermentation Performance

Glucose, ethanol, and SO_2_ tolerances were evaluated for the secondary selection procedure. Each isolate was cultured in YPD liquid medium at 25 °C for 72 h, with glucose concentrations of 100, 200, 300 g/L; ethanol concentrations of 6%, 9%, 12%, 15% (*v*/*v*) for *Saccharomyces cerevisiae* (3%, 5%, 7%, 9% (*v*/*v*) for *non-Saccharomyces cerevisiae*); and SO_2_ concentrations of 100, 150, 200, 300 mg/L for *Saccharomyces cerevisiae* (50, 100, 150, 200 mg/L for *non-Saccharomyces cerevisiae*). Cell growth kinetics were monitored at 600 nm at 12 h.

To evaluate the fermentation performance of the above-screened strains, the purified yeast strains were incubated in a YPD liquid medium at 28 °C for 48 h. When the yeast cell concentrations reached 1 × 10^6^ colony-forming units (CFU)/mL, they were inoculated with simulated mulberry juice [16] and cultured at 25 °C for 14 d. The fermentation was sampled for detection at 0, 2, 4, 6, 10, and 14 d. The °Brix and pH values were detected using a handheld portable device PLA-1 from ATAGO (Japan) and pH meter PHS-25 of Shenzhen Youmi Instrument Equipment Co., Ltd. (Shenzhen, China), respectively, while the glucose, fructose, and glycerol were detected by HPLC [19].

### 2.4. The Practical Fermentation and Aging Treatments with the Selected Strains

We screened the yeast strains with high production of pyruvate acid and acetaldehyde in the initial screening and screened the yeast strains with strong stress resistance and excellent fermentation performance for the subsequent fermentation test of mulberry wine.

The mulberry fruit was selected, squeezed, and packed into a fermentation bottle containing 120 mg/L SO_2_ and 50 mg/L pectinases. After impregnation at 4 °C for 12 h, sucrose was added to adjust the soluble solid contents to 21–22 °Brix. The inoculation strategies of the mulberry must are shown in Table S2. During single fermentation, the initial strain inoculation amount was 1% (*v*/*v*). During the subsequent fermentation, *non-Saccharomyces cerevisiae* was first inoculated at 0.5% (*v*/*v*), and after 3 d, *Saccharomyces cerevisiae* was inoculated at 0.5% (*v*/*v*). All groups were set at 22–25 °C and used °Brix as the fermentation end indicator.

After fermentation, the skin and residue were removed by filtration through gauze, and the liquor was treated with sulfite (120 mg/L) and placed in a bottle. The mulberry wines were aged at 17 °C (cellar temperature), 22 °C (normal temperature), and 37 °C (accelerated storage temperature).

### 2.5. Analysis of Basic Physical and Chemical Indicators of Mulberry Juice and Wine

The basic physical and chemical properties of mulberry wine, including the content of total sugar, ethanol, malic acid, total acid, volatile acid, and total phenolics, were determined according to the following methods [16].

### 2.6. Determination of Total Sugar, Ethanol, and Malic Acid in Mulberry Juice and Mulberry Wine

The content of total sugar, ethanol, and malic acid in mulberry juice and mulberry wine was determined by an automatic wine analyzer (Oeno Foss, Denmark FOSS Co., Ltd. (Hilleroed, Denmark)).

### 2.7. Determination of Total Acidity and Volatile ACID in Mulberry Juice and Wine

Refer to GB/T15038-2006 for determining total acidity and volatile acid in mulberry juice and mulberry wine [20].

### 2.8. Determination of Total Phenolics in Mulberry Juice and Wine

Total phenolic content in mulberry wine was determined by the modified Folin–Ciocalteu method [21]. The results were presented in mg gallic acid equivalent mg/L of mulberry wine sample.

### 2.9. The Detection Methods of Color Index in Mulberry Juice and Wine

#### 2.9.1. Determination of Color Parameters by CIELAB Method

Mulberry wine color was measured using the CIELAB method. Samples were filtered through a 0.45 μm membrane and diluted by 5 times. Absorption was measured at 450, 520, 570, and 630 nm, respectively, with reference to the distilled water. The L*, a*, b*, H*, and C* values were calculated using the formula reported by Pérez-Caballero et al. [22].

#### 2.9.2. Color Fitting by CIELAB Method

The parameters of lightness L*, red/green hue a*, and blue/yellow hue b* were calculated by measuring the color parameters in the experiment and control groups. Color changes were determined by a* and b*, and color-levered changes were determined by L*. Adobe Photoshop software was used to illustrate the results, as shown in the following figure.

### 2.10. Analysis of Total Anthocyanin Content in Mulberry Wine

The total anthocyanin content was determined by the pH-differential method [16].

### 2.11. UPLC-MS/MS for Anthocyanin and Anthocyanin-Derived Compounds

The anthocyanins and anthocyanin-derived were identified using a UPLC-MS/MS (Agilent, Santa Clara, CA, USA), equipped with a Waters ACQUOTY UPLC BEH C18 (2.1 mm × 100 mm, 1.7 μm). The chromatographic conditions were established according to a previous study [16]. Specific conditions were as follows:

The injection volume was 2.0 μL with a 0.2 mL/min flow. The mobile phase consisted of 2 phases: (A) and (B). Mobile phase (A) was 1% (*w*/*w*) formic acid in water, and mobile phase (B) was 1% (*w*/*w*) formic acid in acetonitrile. The elution conditions were as follows: isocratic elution 7% (B), 0 to 3 min; linear gradient from 7% (B) to 30%, 40 min; to 7% (B), 45 min; at 7% (B), 45–48 min. The column temperature was 40 °C, and the detection wavelength was 520 nm.

For the MS analysis. The source parameters were optimized at a spray voltage of 3.2 kV(+)/3.0 kV(−). The other parameters included a capillary temperature of 320 °C, an auxiliary gas heater temperature of 350 °C, a sheath gas pressure of 40 arbs, and an auxiliary gas pressure of 15 arbs. The full MS scan ranged from 100 to 1500 *m*/*z*.

### 2.12. HS-SPME-GC-MS for Volatile Compound Identification

A previously delineated method was used to extract the volatile compounds by HS-SPME-GC-MS [23]. Specific conditions were as follows:

SPME Conditions: A volume of 3 mL of the sample was added to a 10 mL headspace vial, followed by 0.5 g NaCl and 2 μL 2-octanol (internal standard, 0.822 g/L). The mixture was extracted at 40 °C for 50 min using an SPME fiber with a 50/30 μm DVB/CAR/PDMS coating. The analytes were desorbed in the GC-MS injection port for 8 min.

GC Conditions: The analysis was performed using GC-MS (Thermo Fisher, USA). The sample was introduced into the injection port in the splitless mode and injector temperature at 250 °C. The chromatographic separation was achieved on a SUPELCOWAX 10 column (60 m × 0.25 mm, 0.25 μm). The GC oven temperature program started at 50 °C with a hold time of 1 min, followed by an increase at 3 °C/min up to 180 °C, and then ramped at 20 °C/min up to 230 °C, which was held for 15 min.

MS Conditions: Transmission line temperature at 280 °C, Ion source temperature at 230 °C. Full scan mode from 40 to 450 amu.

The compounds were identified by matching their standard spectra with the NIST mass spectral library.

### 2.13. Statistical Analysis

Each test was repeated three times. All the results were expressed as a mean ± standard deviations (SD). IBM SPSS Statistics 23 was applied for statistical and variance (ANOVA) analysis with a significance level of 0.05. Origin Pro 2022 was employed to draw charts, and Photoshop software was used for color fitting.

## 3. Results

### 3.1. Four Strains Were Screened for High Yield of Pyruvate Acid, and Four Strains Were Screened for High Yield of Acetaldehyde

**Figure 1:**
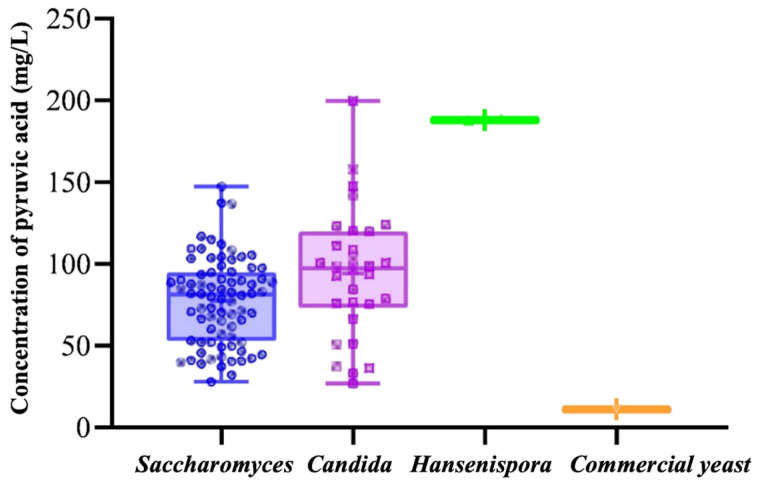
Distribution of pyruvate acid content in fermentation broth among different species.

In this study, the pyruvate acid production capacity of 110 yeast strains was assessed using the deep-hole plate microfermentation method, with commercial yeast AMR-1 (commercial wine yeast) serving as a control. The selected yeast strains exhibited varying abilities to produce pyruvate acid, as depicted in Table 1, which shows the pyruvate acid content in the fermentation broth for each strain. Figure 1 illustrates the distribution of pyruvate acid content among different species of test strains, highlighting variations in pyruvate acid production capabilities across different species or even within different strains of the same genus. Pyruvate acid production of non-*Saccharomyces cerevisiae* strains ranged from 26.93 to 199.63 mg/L, while that of *Saccharomyces cerevisiae* strains ranged from 28.01 to 147.42 mg/L. The pyruvate acid production capacity of the selected 110 strains exceeded that of commercial *Saccharomyces cerevisiae* AMR-1 (11.11–10.76 mg/L). *Candida apicola* I15 from Hebei Province, *Hanseniaspora uvarum* SXC11 from Shanxi Province, *Hanseniaspora uvarum* GS8 from Gansu Province, and *Candida xestobii* D1 from Ningxia Province were the top four strains with the highest pyruvic acid content at levels of 199.63 ± 5.35 mg/L, 188.69 ± 7.92 mg/L, 187.51 ± 1.46 mg/L, and 157.78 ± 5.45 mg/L, respectively. Among the top 20 pyruvate-producing strains, 60% were non-*Saccharomyces cerevisiae*. But, of the last 20 pyruvate-producing strains, 80% were *Saccharomyces cerevisiae*.

**Figure 2:**
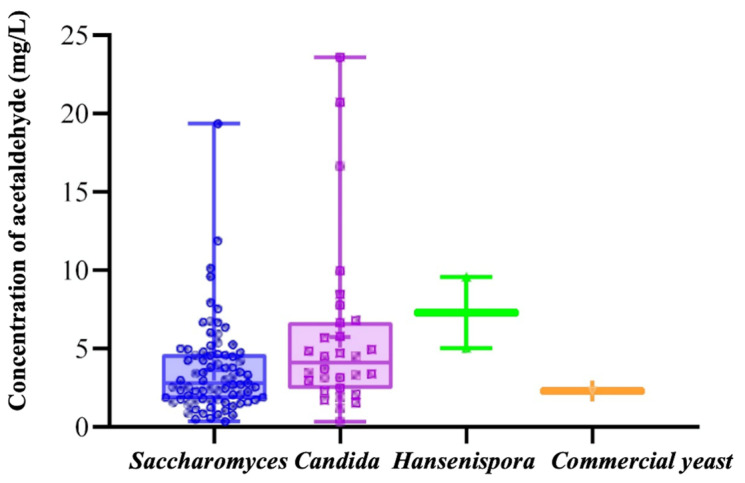
Distribution of acetaldehyde content in fermentation broth among different species.

In this study, the acetaldehyde production capacity of selected yeast strains was evaluated using the deep-hole plate microfermentation method, with AMR-1 serving as a control. The acetaldehyde contents in the fermentation broth of 110 yeast strains and 1 commercial *Saccharomyces cerevisiae* strain are presented in Table 2. The results also demonstrated significant variations in acetaldehyde production among different species and strains within the same genus, which aligns with the findings on pyruvate production by yeast. Among them, 78 strains of *Saccharomyces cerevisiae* produced acetaldehyde ranging from 0.37 to 19.37 mg/L, while non-*Saccharomyces cerevisiae* produced acetaldehyde ranging from 0.34 to 23.60 mg/L (Figure 2). The top four strains exhibiting high acetaldehyde production were *Candida globrata* GS13, *Candida globrata* GS30, *Saccharomyces cerevisiae* GS32, and *Candida globrata* GS31 from Gansu province, respectively, with yields of 23.60 ±2.43 mg/L, 20.71 ± 2.82 mg/L, 19.37 ± 4.29 mg/L, and 16.65 ± 0.54 mg/L, respectively. In addition, among the top 20 pyruvate-producing strains, 55% were *non-Saccharomyces cerevisiae*, a pattern that differs from pyruvate acid production rules.

Four yeast strains with high-yield pyruvate acid (I15, SXC11, GS8, and D1) and four yeast strains with high-yield acetaldehyde (GS13, GS30, GS31, and GS32) were initially screened out via deep well plate microcultures. The relatively low fermentative power of most *non-Saccharomyces* yeast prevents them from complete independent fermentation. Therefore, these eight strains were evaluated in simulated mulberry juice to assess the feasibility of practical mulberry wine fermentation.

### 3.2. Three Strains Were Selected Based on Fermentation Characteristics and Stress Resistance

The growth of *Saccharomyces cerevisiae* GS32 and *non-Saccharomyces cerevisiae* strains SXC11, GS8, GS30, GS31, GS13, I15, and D1 was measured in this experiment under varying glucose concentrations. The results are presented in Figure S1. All eight yeast strains exhibited growth and reproduction capabilities across different glucose concentrations. Notably, *Saccharomyces cerevisiae* GS32 (Figure S1A) demonstrated the highest resistance to hyperosmotic pressure among all tested strains. In contrast to *Saccharomyces cerevisiae*, *non-Saccharomyces cerevisiae* displayed distinct growth patterns; both GS13 (Figure S1E) and I15 (Figure S1G) were inhibited at various glucose concentrations. SXC11 (Figure S1B), GS30 (Figure S1D), GS13 (Figure S1F), and D1 (Figure S1H) exhibited optimal growth conditions when the sugar concentration was set at 100 g/L while the growth of GS8 (Figure S1C) was hindered at a sugar concentration of 300 g/L. Overall, *Saccharomyces cerevisiae* were able to withstand higher osmotic stress from high glucose concentration than *non-Saccharomyces cerevisiae*.

The growth of *Saccharomyces cerevisiae* GS32 and *non-Saccharomyces cerevisiae* SXC11, GS8, GS30, GS31, I15, and D1 was measured in this experiment under varying ethanol concentrations. The results are presented in Figure S2. *Saccharomyces cerevisiae* GS32 (Figure S2A) exhibited inhibition but still demonstrated normal growth and reproduction at an ethanol concentration of 12% (*v*/*v*), while complete cessation occurred at 15% (*v*/*v*). *Non-Saccharomyces cerevisiae* strains GS13 (Figure S2F) and D1 (Figure S2H) were able to grow by reproducing across different ethanol concentrations. Under conditions of 9% (*v*/*v*), both GS30 (Figure S2D) and I15 (Figure S2G) displayed robust growth; however, they were also capable of growing and reproducing within the range of 3% to 7% (*v*/*v*). SXC11 (Figure S2B) and GS8 (Figure S2C) experienced stagnation between the ethanol concentrations of 7 and 9% (*v*/*v*). Conversely, born under conditions ranging from 3 to 7% (*v*/*v*), GS31 (Figure S2E) exhibited inhibition.

The growth of *Saccharomyces cerevisiae* GS32 and *non-Saccharomyces cerevisiae* strains SXC11, GS8, GS30, GS31, I15, and D1 was measured in this experiment under varying concentrations of SO_2_. The results are presented in Figure S3. *Saccharomyces cerevisiae* GS32 (Figure S3A) exhibited a high tolerance to SO_2_. Except for strain I15, the growth of *non-Saccharomyces cerevisiae* strains was significantly inhibited at all tested levels of SO_2_ concentration, indicating their high sensitivity towards SO_2_ (Figure S3B–H).

**Figure 3:**
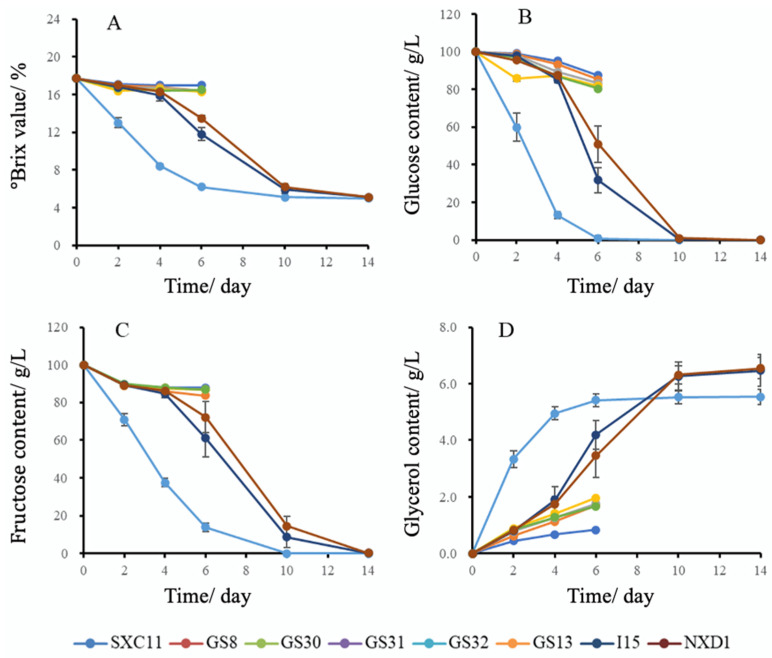
Changes in °Brix, glucose, fructose, and glycerol during fermentation of 8 yeast strains: (**A**) °Brix; (**B**) glucose; (**C**) fructose; (**D**) glycerol.

*Saccharomyces cerevisiae* GS32 and *non-Saccharomyces cerevisiae* strains SXC11, GS8, GS30, GS31, GS13, I15, and D1 were individually inoculated into simulated mulberry juice. As observed from the changes in °Brix value (Figure 3A), *Saccharomyces cerevisiae* GS32 initiated fermentation rapidly after yeast addition and completed fermentation on the 14th day. Conversely, the °Brix value of *non-Saccharomyces cerevisiae* strains showed minimal change during the first 4 days. Fermentation for I15 and D1 started rapidly on the 6th day and was completed by the 14th day. However, after inoculation for 6 days, the °Brix value of the other five *non-Saccharomyces cerevisiae* strains tended to stabilize, indicating their inability to independently complete alcohol fermentation and requiring participation from *Saccharomyces cerevisiae* to fully utilize sugar conversion into alcohol.

The results regarding reducing sugar and glycerol production capacity of these eight strains are presented in Figure 3B–D. It can be observed from Figure 3B,C that glucose and fructose variations during fermentation follow a similar trend as that of °Brix value. Although both sugars decrease throughout the consumption process, glucose is initially consumed at the beginning of fermentation until completely depleted, subsequently followed by a rapid reduction in fructose content. The changes in glycerol content among all eight yeast strains during fermentation are depicted in Figure 3D. Three yeast strains (GS32, I15, D1) exhibited an initial increase followed by stabilization in glycerol levels with maximum content ranging between 5.42 and 6.55 g/L. In contrast, the remaining five strains that did not complete fermentation had glycerol production.

Combined with the ability to produce pyruvate acid and acetaldehyde and their oenological tolerance, *Saccharomyces* GS32, *Candida* GS13, and *Candida* D1 are recommended for use in mulberry wine fermentation.

### 3.3. GS32 and D1 Significantly Improved the Total Anthocyanin Concentration and Color Stability of Mulberry Wine

**Figure 4:**
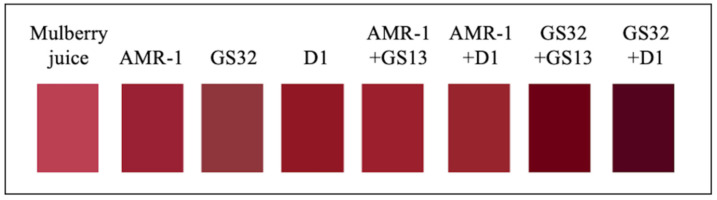
The color-fitting diagram of mulberry juice and mulberry wine.

These strains were further tested for making mulberry wine. The fermented and aged wines were evaluated for color preservation, anthocyanin stability, and other important physical and chemical properties (Table S3). The parameter changes in mulberry juice and mulberry wine CIELAB are shown in Table S4. L* indicates the color brightness of mulberry wine, and the larger the value, the brighter the color. After fermentation, compared with mulberry juice, the L* value of mulberry wine decreases, and the brightness decreases. a* indicates the reddish-green degree of mulberry wine, and the larger the value, the more red color. Only the a* of GS32 + D1 group in mulberry wine has a significant difference with mulberry juice. b* represents the degree of yellow blue of mulberry wine, and the larger the value, the more yellow hue. In the treatment group, only the b* value of the GS32 + D1 group decreased, the blue hue increased, and the b* value of the other groups increased. Figure 4 more intuitively shows the results of Table S4, GS32 + D1 group mulberry wine color is a darker, darker purple.

The impact of high-yield pyruvate acid and acetaldehyde strains on the volatile components of the mulberry wine was also analyzed by GC-MS. A total of 57 different aroma compounds were detected (Table S6). The aroma of samples fermented by different yeast groups was also different.

**Figure 5:**
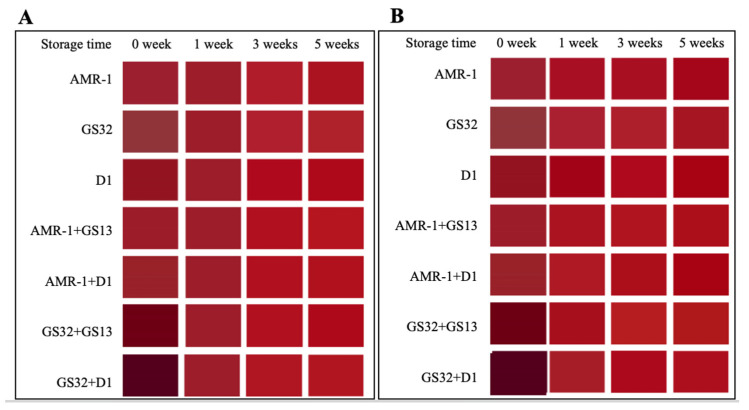

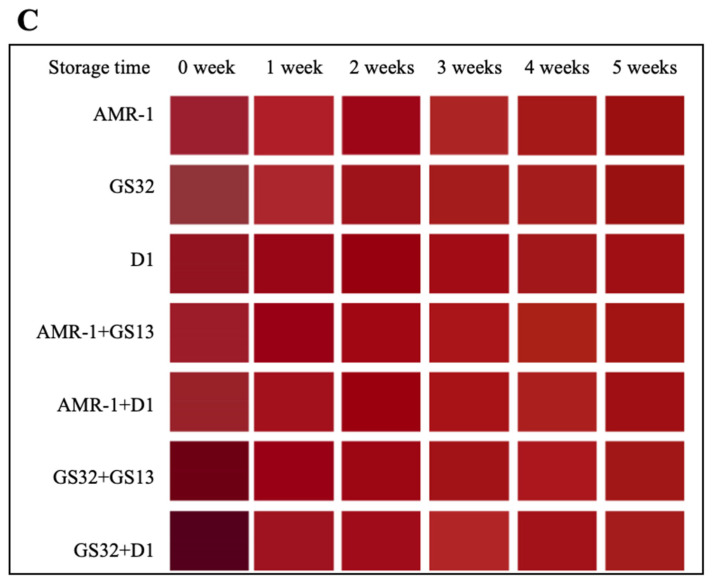
Color fitting diagram of mulberry during aging. (**A**) Color fitting diagram of mulberry wine aged at 17 °C; (**B**) Color fitting diagram of mulberry wine aged at 22 °C; (**C**) Color fitting diagram of mulberry wine aged at 37 °C.

Fruit wine aging is an important part of the process of fruit wine brewing, which plays an important role in the quality of fruit wine. In this experiment, mulberry wine was aged under the conditions of 17 °C, 22 °C, and 37 °C, and the changes in CIELAB parameters of mulberry wine were observed, respectively (as shown in Table S5). Under the condition of 17 °C, L*, a*, b*, C*, and H* values of mulberry wine increased from 0 to 1 week of aging; that is, color brightness increased, purple hue weakened, red hue enhanced, yellow hue enhanced, color saturation and gloss enhanced. With the extension of aging time, the CIELAB parameters of each treatment group did not change significantly. The variation in parameters under condition 22 °C is similar to that under condition 17 °C. The color of mulberry wine aged for 1 week under the condition of 22 °C (Figure 5B) was close to that aged for 3 weeks under the condition of 17 °C (Figure 5A), and there was no significant change in the color of mulberry wine in each treatment group with the extension of aging time. Under 37 °C accelerated aging (Figure 5C), the L* value and brightness of mulberry wine decreased after 5 weeks of aging, except for the GS32 + D1 group; The a* value increased, and the red hue increased. The b* value increased, and the yellow hue increased. It may be that the yeast strains that are high in pyruvate acid and acetaldehyde convert with monomer anthocyanins to vitisins with orange hues, resulting in a change in color.

After 5 weeks of aging, L*, a*, b*, and C* values of mulberry wine decreased with the increase in temperature, which may be because the stability of anthocyanins gradually decreased with the increase in temperature. However, there was no significant difference in the color of mulberry wine in each treatment group after 1 to 5 weeks of aging, which may be due to the stabilizing effect of yeast strains with high pyruvate acid and acetaldehyde production on mulberry wine color.

### 3.4. GS32 Improve Total Anthocyanins (TA) Concentration After Fermentation, and Slow TA, C3G and C3R Degradation During Aging

**Figure 6:**
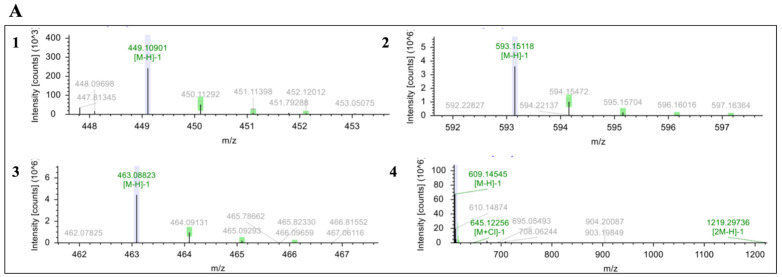

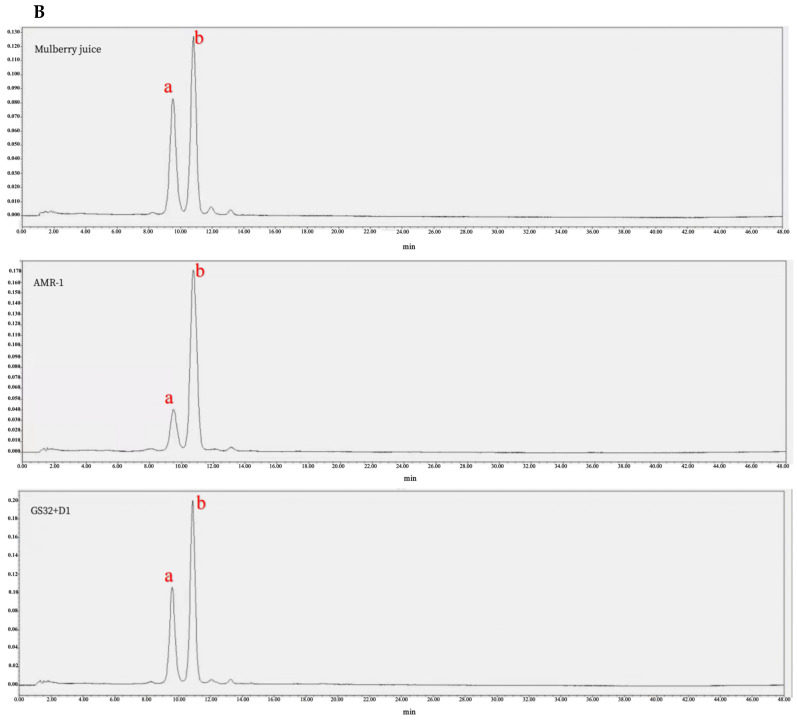
Identification information of anthocyanins and anthocyanin derivatives in mulberry juice and mulberry wine by UPLC-MS: (**A**) Mass spectra of anthocyanin in mulberry wine. 1: Centaurin-3-O-glucoside (C3G); 2: Centaurin-3-O-rutinoside (C3R); 3: Pelargonin-3-O-glucoside (P3G); 4: Pelargonin-3-O-rutinoside (P3R). (**B**) UPLC chromatogram of mulberry juice and mulberry wine. a: C3G; b: C3R.

In order to further clarify whether the effect of yeast strains with high pyruvate acid and acetaldehyde on the color stability of mulberry wine was due to the reaction of monomeric anthocyanins with small molecular substances (pyruvate acid and acetaldehyde) to form pyranoanthocyanins [24,25,26], UPLC-MS was used in this experiment to identify anthocyanins and anthocyanin derivatives in mulberry juice and mulberry wine. The results are shown in Figure 6. However, the corresponding anthocyanin derivatives were not detected when UPLC-MS was used to detect the mulberry wine at the end of fermentation.

The contents of total anthocyanins, C3G, and C3R in mulberry juice and mulberry wine are shown in Table 3. Mulberry anthocyanins were leached from mulberry peel residues during the brewing process of mulberry wine, but the retention rate of anthocyanins in experimental groups was different after fermentation. The contents of total anthocyanins in GS32 and D1 single-strain fermentation groups were the highest, 901.70 ± 56.52 mg/L and 866.02 ± 28.79 mg/L, respectively. However, the total anthocyanin content of the GS32 + D1 mixed yeast fermentation treatment group was 846.27 ± 77.40 mg/L, which was lower than that of the single strain fermentation group, indicating that the fermentation mode of strain had a certain effect on the anthocyanin content of mulberry wine. This speculation has also been reported in previous studies, which have previously shown that different microbial community structures or different inoculation methods (e.g., simultaneous inoculation or sequential inoculation) can affect the color of wine [27,28]. As for the monomer anthocyanins, the C3G content showed a decreasing trend after fermentation, which may be because the monomer anthocyanins reacted with metabolites produced by yeast to form more stable polymers. In recent years, more and more studies have shown that the use of yeast metabolites during the fermentation process promotes the formation of more structurally stable anthocyanin derivatives from monomeric anthocyanins, thus improving the color of fruit wines. This has led to increased interest in this biological approach [16,29]. The decrease rate of C3G in the GS32 treatment group was the highest, from 240.85 ± 0.36 mg/L to 151.39 ± 4.47 mg/L. However, the mixed fermentation of the GS32 strain with the GS13 or D1 strain slowed down the reduction in C3G. In contrast to C3G, C3R content showed an increasing trend after fermentation, and the C3R content increased the most in the AMR-1 control group (48.9%), followed by GS32 and D1.

**Figure 7:**
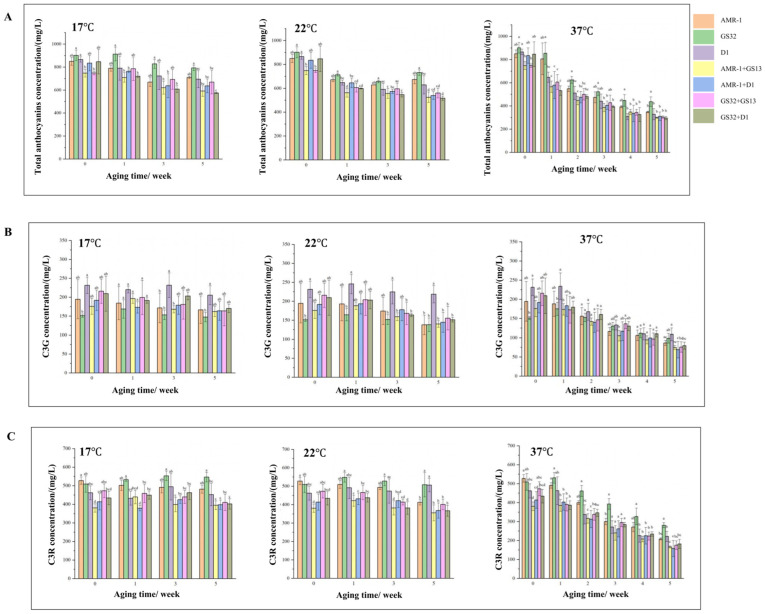
Changes in total anthocyanins, C3G, and C3R in mulberry wine during aging: (**A**) changes in total anthocyanins in mulberry wine at 17 °C, 22 °C, and 37 °C during aging; (**B**) changes in C3G in mulberry wine at 17 °C, 22 °C, and 37 °C during aging; (**C**) changes in C3G in mulberry wine at 17 °C, 22 °C, and 37 °C during aging. Different letters indicated significant differences in the index, *p* < 0.05.

The content changes in total anthocyanins, C3G, and C3R in mulberry wine during aging are shown in Figure 7. The degradation rates of anthocyanins increased with the increase in temperature. At the end of aging, the degradation rates of total anthocyanins in the GS32 group were 12.09%, 18.91%, and 51.52% under the conditions of 17 °C, 22 °C, and 37 °C, respectively, which were lower than those in the control group (16.30%, 20.71% and 59.05% in the control group, respectively). In the accelerated aging test, the final total anthocyanin residue in the GS32 group was higher than that in other treatment groups and the control group. The degradation rate of C3G was generally higher than that of C3R under the aging conditions of 17 °C and 22 °C, which indicated that C3R had higher stability and was more difficult to decompose than C3G in the aging process. Under the accelerated aging condition of 37 °C, the degradation rates of C3G and C3R in the GS32 treatment group were 34.91% and 44.92%, respectively, which were lower than those in other treatment groups and the control group. The total amount of C3G and C3R in the mulberry wine of the GS32 group was the highest. According to the changes in total anthocyanins, C3G, and C3R during aging, all indexes of the GS32 treatment group were better than the other six groups. These results indicated that the yeast strain GS32 could improve the stability of the anthocyanins of mulberry. However, the mixed fermentation of GS32 with other strains may weaken its ability to stabilize anthocyanins.

## 4. Discussion

China has a vast territory, a complex natural environment, and significant differences in climate and soil conditions, which lead to differences in the types and characteristics of native wild yeast. In this experiment, the content of pyruvate acid and acetaldehyde produced between different species and between the same producing area and different producing areas were significantly different. Romano et al. [17] studied the acetaldehyde production capacity of *Saccharomyces cerevisiae* screened from different grape varieties in different regions of Italy, and the acetaldehyde production capacity varied among different species. The content of high-producing acetaldehyde strains could reach more than 50 mg/L, while the content of low-producing acetaldehyde strains could reach less than 20 mg/L. This was similar to the results of this study, where the content of acetaldehyde in the fermentation broth of 110 strains in this experiment was between 0.34 and 23.60 mg/L. Li et al. [30] found that the acetaldehyde production of *non-Saccharomyces cerevisiae,* except *Schizosaccharomyces prombe* was significantly lower than that of *Saccharomyces cerevisiae*. In this experiment, there is only one *Saccharomyces cerevisiae* of the top three strains producing acetaldehyde. But this also shows that the acetaldehyde production capacity of different strains is different. In the study of Morata et al. [31], the pyruvate production content of 10 strains of *Saccharomyces cerevisiae* ranged from 60 to 132 mg/L, indicating that different strains had different pyruvate acid production capabilities. The pyruvate acid yield of the selected yeast strains in this experiment was between 26.93 and 199.63 mg/L. It can be seen that strains of different species have different pyruvate acid production capabilities. There are abundant wild yeast resources in China, and the production capacity of pyruvate acid and acetaldehyde varies greatly between different regions and different species. The screening of high-yielding pyruvate acid yeast combined with the brewing characteristics of the strain itself may provide an important way to use wild yeast resources in mulberry wine with regional characteristics and long-term color stability.

During the whole experiment period, the anthocyanins stability of mulberry wine could be improved, and the color of mulberry wine could be maintained. However, we did not detect vitisins anthocyanins corresponding to C3G and C3R in any of the treatment groups in the present experiment, probably because vitisins anthocyanins formation was affected by the pH of the substrate. Vitisins A is an adduct derived from the reaction of anthocyanins with enolated pyruvate acid. The formation mechanism of vitisins A is as follows: under certain acidic conditions, the pyruvate undergoes carbonyl enolization, the electronegative methyl group and the C4 and C5 hydroxyl group of anthocyanins undergo addition condensation, and then a new pyranoid ring is formed by dehydration and oxidation [32]. By combining C3G, C3R, Cyanidin-3-O-Sophoroside, and Cyanidin-3-O-Sambubioside extracted from blackberry, sweet cherry, raspberry, and elderberry with pyruvate in simulated solutions with different pH values (1.0–7.0) to generate polymers, Oliveria et al. [33] found that the maximum yields were obtained at pH 1.0 and 2.0. From pH 3.0 to 7.0, the amount of polymer produced gradually decreased with the increase in pH. In this experiment, the pH of mulberry juice was 3.67, and the pH of mulberry wine increased slightly after fermentation, ranging from 3.7 to 3.9. And the real mulberry juice is more complex than the simulated solution, so there are more factors affecting the formation of vitisins.

## 5. Conclusions

In this study, the production capacity of pyruvate acid and acetaldehyde by 78 *Saccharomyces cerevisiae* strains and 32 *non-Saccharomyces cerevisiae* strains in the strain bank of our research group was measured, and four high-yielding pyruvate acid yeast strains were selected—*Candida apicola* I15, *Candida xestobii* D1, *Hanseniaspora uvarum* SXC11, *Hanseniaspora uvarum* GS8, as well as four high-yielding acetaldehyde yeast strains—*Candida globrata* GS13, *Candida globrata* GS30, *Candida globrata* GS31 and *Saccharomyces cerevisiae* GS32. On this basis, the tolerance and fermentation characteristics of eight strains of yeast were further determined. Finally, *Saccharomyces cerevisiae* GS32 and *non-Saccharomyces cerevisiae* D1 and GS13 were selected as yeasts with high acetaldehyde and pyruvate acid production and excellent fermentation performance for subsequent fermentation tests of mulberry wine. Their effects on improving anthocyanin stability and color stability of mulberry wine were further explored. Different strains and inoculation methods would affect the total anthocyanins in mulberry wine. Under the conditions of three aging temperatures (17 °C, 22 °C, and 37 °C), the degradation rates of total anthocyanins, C3G, and C3R in mulberry wine in the GS32 group were the smallest. By fitting the color of mulberry wine during the aging period, it was found that 17 °C could better delay the color transformation of mulberry wine, followed by 22 °C, and finally 37 °C.

## Figures and Tables

**Table 1:** Concentration of pyruvic acid in yeast fermentation broth.

Identification Number	Strain Generus	Pyruvate Acid Concentration (mg/L)
I15	*Candida*	199.63 ± 5.35
D1	*Candida*	157.78 ± 5.45
LN10	*Candida*	147.68 ± 8.61
LN22	*Candida*	141.73 ± 7.68
D18	*Candida*	124.12 ± 7.00
GS18	*Candida*	123.25 ± 0.89
GS10	*Candida*	120.22 ± 6.36
GS31	*Candida*	119.92 ± 6.67
YT4	*Candida*	111.06 ± 3.43
BG3	*Candida*	108.58 ± 0.31
GS16	*Candida*	102.30 ± 7.74
D16	*Candida*	100.70 ± 3.48
GS20	*Candida*	100.70 ± 7.45
GS13	*Candida*	98.83 ± 6.71
GS27	*Candida*	98.39 ± 6.66
LN9	*Candida*	96.25 ± 4.41
LN12	*Candida*	93.67 ± 2.87
YT25	*Candida*	92.54 ± 0.41
GS15	*Candida*	84.58 ± 3.43
GS14	*Candida*	78.79 ± 2.35
F19	*Candida*	76.66 ± 5.36
LN13	*Candida*	75.94 ± 5.26
YT2	*Candida*	75.44 ± 3.00
GS11	*Candida*	66.45 ± 6.30
GS30	*Candida*	51.22 ± 5.03
YT10	*Candida*	50.76 ± 5.21
GS12	*Candida*	37.40 ± 1.28
GS22	*Candida*	36.45 ± 3.73
YT3	*Candida*	33.25 ± 6.02
D40	*Candida*	26.93 ± 5.44
SXC11	*Hanseniaspora*	188.69 ± 7.92
GS8	*Hanseniaspora*	187.51 ± 1.46
GS39	*Saccharomyces*	147.42 ± 5.41
D30	*Saccharomyces*	137.32 ± 7.16
CL16	*Saccharomyces*	136.75 ± 6.87
GS40	*Saccharomyces*	117.01 ± 5.08
SH29	*Saccharomyces*	115.17 ± 3.33
AC29	*Saccharomyces*	112.20 ± 3.44
D22	*Saccharomyces*	109.45 ± 6.14
B47	*Saccharomyces*	109.44 ± 6.81
BH8	*Saccharomyces*	108.39 ± 5.89
XJA1	*Saccharomyces*	105.59 ± 4.46
B43	*Saccharomyces*	104.49 ± 1.04
I43	*Saccharomyces*	104.46 ± 6.66
XJA8	*Saccharomyces*	103.86 ± 0.75
B38	*Saccharomyces*	103.41 ± 4.76
SC37	*Saccharomyces*	102.87 ± 4.29
E68	*Saccharomyces*	99.10 ± 3.07
H68	*Saccharomyces*	97.86 ± 9.50
GS9	*Saccharomyces*	97.54 ± 2.90
E67	*Saccharomyces*	95.22 ± 6.16
SXC9	*Saccharomyces*	94.91 ± 9.45
E63	*Saccharomyces*	93.57 ± 0.97
LH21	*Saccharomyces*	90.90 ± 2.89
GS32	*Saccharomyces*	90.70 ± 7.03
SXC8	*Saccharomyces*	90.31 ± 4.09
D35	*Saccharomyces*	89.95 ± 5.89
YT28	*Saccharomyces*	88.91 ± 6.12
CL1	*Saccharomyces*	88.81 ± 4.82
H66	*Saccharomyces*	88.78 ± 1.49
NC3	*Saccharomyces*	87.90 ± 4.18
H63	*Saccharomyces*	87.53 ± 5.58
CL34	*Saccharomyces*	87.43 ± 6.04
D39	*Saccharomyces*	85.84 ± 3.94
D28	*Saccharomyces*	84.90 ± 5.48
D20	*Saccharomyces*	84.45 ± 1.90
AH39	*Saccharomyces*	83.11 ± 5.34
GS5	*Saccharomyces*	82.76 ± 4.01
E19	*Saccharomyces*	81.90 ± 6.33
E74	*Saccharomyces*	81.88 ± 8.90
E58	*Saccharomyces*	81.79 ± 8.45
GS29	*Saccharomyces*	80.98 ± 2.06
B49	*Saccharomyces*	80.09 ± 7.21
D27	*Saccharomyces*	78.92 ± 7.12
LN21	*Saccharomyces*	77.09 ± 6.46
CL19	*Saccharomyces*	73.16 ± 9.66
E65	*Saccharomyces*	73.12 ± 0.96
E12	*Saccharomyces*	71.60 ± 5.02
GS33	*Saccharomyces*	71.00 ± 2.23
CL14	*Saccharomyces*	70.58 ± 5.09
SXC13	*Saccharomyces*	70.02 ± 6.31
E77	*Saccharomyces*	69.48 ± 2.35
XJA2	*Saccharomyces*	67.82 ± 7.55
B50	*Saccharomyces*	66.62 ± 8.70
BH33	*Saccharomyces*	65.93 ± 2.48
NW9	*Saccharomyces*	65.23 ± 6.24
B46	*Saccharomyces*	61.84 ± 8.24
CL7	*Saccharomyces*	60.24 ± 2.22
SXC6	*Saccharomyces*	57.27 ± 5.97
L59	*Saccharomyces*	55.25 ± 2.87
LH39	*Saccharomyces*	53.33 ± 3.88
LB1	*Saccharomyces*	52.25 ± 5.81
D41	*Saccharomyces*	52.20 ± 1.21
I61	*Saccharomyces*	52.11 ± 4.71
B42	*Saccharomyces*	49.85 ± 1.19
CL8	*Saccharomyces*	49.31 ± 5.60
I42	*Saccharomyces*	46.83 ± 3.21
CL10	*Saccharomyces*	45.74 ± 2.34
NC1	*Saccharomyces*	44.77 ± 2.89
I52	*Saccharomyces*	43.37 ± 4.00
I34	*Saccharomyces*	42.49 ± 6.91
H70	*Saccharomyces*	41.81 ± 4.88
NW12	*Saccharomyces*	41.05 ± 2.60
CL27	*Saccharomyces*	40.79 ± 2.85
I68	*Saccharomyces*	40.52 ± 4.28
H58	*Saccharomyces*	39.99 ± 3.22
YT13	*Saccharomyces*	39.15 ± 5.85
CL26	*Saccharomyces*	37.42 ± 5.23
E11	*Saccharomyces*	32.12 ± 2.80
I56	*Saccharomyces*	28.01 ± 8.45
AMR-1 *	*Saccharomyces*	11.11 ± 0.76

Note: “*” indicates that AMR-1 is commercial *Saccharomyces cerevisiae* and was used as a control.

**Table 2:** Concentration of acetaldehyde in yeast fermentation broth.

Identification Number	Strain Generus	Acetaldehyde Concentration (mg/L)
GS13	*Candida*	23.60 ± 2.43
GS30	*Candida*	20.71 ± 2.82
GS31	*Candida*	16.65 ± 0.54
LN22	*Candida*	9.96 ± 1.99
D1	*Candida*	8.46 ± 0.89
GS14	*Candida*	7.79 ± 1.27
D18	*Candida*	6.81 ± 0.04
LN12	*Candida*	6.66 ± 0.45
GS16	*Candida*	5.78 ± 0.79
I15	*Candida*	5.69 ± 2.85
GS10	*Candida*	4.94 ± 0.62
YT4	*Candida*	4.85 ± 0.98
GS20	*Candida*	4.71 ± 1.00
LN9	*Candida*	4.52 ± 0.56
YT25	*Candida*	4.52 ± 1.05
F19	*Candida*	3.69 ± 0.61
GS15	*Candida*	3.46 ± 0.70
GS12	*Candida*	3.39 ± 0.41
LN13	*Candida*	3.33 ± 0.40
BG3	*Candida*	3.16 ± 0.77
YT3	*Candida*	3.14 ± 0.65
YT2	*Candida*	2.94 ± 0.46
D16	*Candida*	2.48 ± 0.59
YT10	*Candida*	2.28 ± 0.76
GS18	*Candida*	2.09 ± 0.45
GS11	*Candida*	1.88 ± 0.34
LN10	*Candida*	1.72 ± 0.42
GS27	*Candida*	1.53 ± 0.13
GS22	*Candida*	1.20 ± 0.11
D40	*Candida*	0.34 ± 0.11
SXC11	*Hanseniaspora*	9.58 ± 0.13
GS8	*Hanseniaspora*	5.04 ± 0.67
GS32	*Saccharomyces*	19.37 ± 4.29
D35	*Saccharomyces*	11.88 ± 2.15
LN21	*Saccharomyces*	6.04 ± 0.90
H68	*Saccharomyces*	7.56 ± 0.86
CL1	*Saccharomyces*	6.76 ± 2.43
LH39	*Saccharomyces*	6.67 ± 1.00
CL10	*Saccharomyces*	6.37 ± 0.40
SXC8	*Saccharomyces*	5.95 ± 0.54
D27	*Saccharomyces*	5.26 ± 1.10
GS39	*Saccharomyces*	4.97 ± 0.40
E74	*Saccharomyces*	4.76 ± 1.29
E68	*Saccharomyces*	4.66 ± 1.94
B38	*Saccharomyces*	4.58 ± 1.53
SXC13	*Saccharomyces*	4.50 ± 0.62
D41	*Saccharomyces*	4.25 ± 0.36
CL19	*Saccharomyces*	4.02 ± 0.61
SXC6	*Saccharomyces*	3.81 ± 0.64
AH39	*Saccharomyces*	10.13 ± 0.58
NW9	*Saccharomyces*	9.61 ± 0.39
B43	*Saccharomyces*	6.70 ± 1.25
L59	*Saccharomyces*	6.04 ± 0.90
D30	*Saccharomyces*	5.38 ± 0.56
I43	*Saccharomyces*	5.19 ± 0.72
BH8	*Saccharomyces*	5.02 ± 1.92
E12	*Saccharomyces*	4.81 ± 0.05
SXC9	*Saccharomyces*	4.59 ± 0.02
BH33	*Saccharomyces*	4.57 ± 1.96
NC3	*Saccharomyces*	4.26 ± 0.17
H63	*Saccharomyces*	4.22 ± 1.05
I52	*Saccharomyces*	3.82 ± 0.31
B46	*Saccharomyces*	3.78 ± 0.96
E63	*Saccharomyces*	3.52 ± 0.98
D22	*Saccharomyces*	3.49 ± 0.55
B47	*Saccharomyces*	3.43 ± 1.39
I42	*Saccharomyces*	3.35 ± 0.53
SC37	*Saccharomyces*	3.24 ± 0.15
CL27	*Saccharomyces*	2.98 ± 0.47
CL14	*Saccharomyces*	2.71 ± 0.56
I34	*Saccharomyces*	2.58 ± 0.78
XJA8	*Saccharomyces*	2.55 ± 0.71
CL34	*Saccharomyces*	2.46 ± 0.45
H58	*Saccharomyces*	2.36 ± 0.57
AC29	*Saccharomyces*	2.33 ± 0.81
H70	*Saccharomyces*	2.08 ± 0.66
E77	*Saccharomyces*	1.90 ± 0.33
YT28	*Saccharomyces*	1.78 ± 0.27
I61	*Saccharomyces*	1.74 ± 0.19
D28	*Saccharomyces*	1.58 ± 0.52
GS29	*Saccharomyces*	1.44 ± 0.10
GS33	*Saccharomyces*	1.23 ± 0.07
B50	*Saccharomyces*	1.14 ± 0.13
E67	*Saccharomyces*	0.89 ± 0.24
SH29	*Saccharomyces*	0.81 ± 0.27
E58	*Saccharomyces*	0.57 ± 0.18
CL16	*Saccharomyces*	0.37 ± 0.21
CL7	*Saccharomyces*	3.04 ± 0.33
CL8	*Saccharomyces*	2.96 ± 0.51
I68	*Saccharomyces*	2.81 ± 0.36
XJA2	*Saccharomyces*	2.70 ± 0.03
E65	*Saccharomyces*	2.56 ± 0.45
NC1	*Saccharomyces*	2.42 ± 0.59
I56	*Saccharomyces*	2.34 ± 0.55
CL26	*Saccharomyces*	2.31 ± 0.40
D39	*Saccharomyces*	2.27 ± 0.20
D20	*Saccharomyces*	2.08 ± 0.20
B49	*Saccharomyces*	2.04 ± 0.28
GS9	*Saccharomyces*	1.89 ± 0.22
GS5	*Saccharomyces*	1.80 ± 0.25
GS40	*Saccharomyces*	1.76 ± 0.14
E19	*Saccharomyces*	1.72 ± 0.05
LH21	*Saccharomyces*	1.60 ± 0.36
LB1	*Saccharomyces*	1.57 ± 0.16
NW12	*Saccharomyces*	1.51 ± 0.36
XJA1	*Saccharomyces*	1.32 ± 0.23
YT13	*Saccharomyces*	1.05 ± 0.16
B42	*Saccharomyces*	0.86 ± 0.19
H66	*Saccharomyces*	0.79 ± 0.20
E11	*Saccharomyces*	0.52 ± 0.04
AMR-1 *	*Saccharomyces*	2.32 ± 0.25

Note: “*” indicates that AMR-1 is commercial *Saccharomyces cerevisiae* and was used as a control.

**Table 3:** Anthocyanin content in mulberry juice and mulberry wine.

	Mulberry Juice	Mulberry Wine						
		AMR-1 (CK)	GS32	D1	AMR-1 + GS13	AMR-1 + D1	GS32 + GS13	GS32 + D1
Total anthocyanins (mg/L)	753.67 ± 47.00 ^b^	849.36 ± 41.21 ^ab^	901.70 ± 56.52 ^a^	866.02 ± 28.79 ^a^	747.93 ± 41.56 ^b^	834.24 ± 81.05 ^ab^	748.11 ± 19.45 ^b^	846.27 ± 77.40 ^ab^
C3G (mg/L)	240.85 ± 0.36 ^a^	194.73 ± 51.89 ^ab^	151.39 ± 4.47 ^b^	231.47 ± 21.71 ^a^	175.86 ± 20.99 ^ab^	191.81 ± 27.08 ^ab^	215.78 ± 31.91 ^ab^	209.43 ± 46.33 ^ab^
C3R (mg/L)	354.61 ± 18.72 ^d^	528.01 ± 19.81 ^a^	509.29 ± 43.95 ^a^	462.07 ± 37.21 ^ab^	380.87 ± 22.60 ^cd^	413.72 ± 43.90 ^bcd^	473.09 ± 36.89 ^ab^	434.09 ± 36.04 ^bc^

Note: Different letter superscripts in the same row of the table indicate that there are significant differences in this index, *p* < 0.05; CK stands for contrast.

## Data Availability

The data that support the findings of this study are available from the corresponding author upon reasonable request.

## Supplementary Materials

The following supporting information can be downloaded at: https://www.mdpi.com/article/10.3390/foods14132278/s1, Figure S1: Growth curve of 8 yeast strains under different sugar concentrations. (A) GS32 strain; (B) SXC11 strain; (C) GS8 strain; (D) GS30 strain; (E) GS31 strain; (F) GS13 strain; (G) I15 strain; (H) D1 strain; Figure S2: Growth curve of 8 yeast strains under different alcohol concentrations. (A) GS32 strain; (B) SXC11 strain; (C) GS8 strain; (D) GS30 strain; (E) GS31 strain; (F) GS13 strain; (G) I15 strain; (H) D1 strain; Figure S3: Growth curve of 8 yeast strains under different SO2 concentrations. (A) GS32 strain; (B) SXC11 strain; (C) GS8 strain; (D) GS30 strain; (E) GS31 strain; (F) GS13 strain; (G) I15 strain; (H) D1 strain; Table S1: The information of 110 strains of yeasts used in the experiments; Table S2: Mulberry wine fermentation treatment group; Table S3: The results of indexes of mulberry juice and mulberry wine; Table S4: CIELab color parameters of mulberry juice and mulberry wine; Table S5: Summary of CIELab color parameter in mulberry wine during aging; Table S6: Content of volatile components in mulberry wine.
