# Supplementary material for: Screening for High-Yielding Pyruvate and Acetaldehyde Yeasts and Their Application in Improving the Stability of Anthocyanin in Mulberry Wine
Source: Foods. 2025 Jun 27;14(13):2278. doi: 10.3390/foods14132278 (PMC12248806; doi:10.3390/foods14132278)
Supplement: Supplementary file 1 [file foods-14-02278-s001.zip › foods-3621209-SI.pdf]

Supplementary Materials

Table S1 The information of 110 strains of yeasts used in the experiments

Strain source	Identification number	Strain generus	Strain species
Hei Bei	I34	*Saccharomyces*	*Saccharomyces cerevisiae*
	I42		*Saccharomyces cerevisiae*
	I43		*Saccharomyces cerevisiae*
	I52		*Saccharomyces cerevisiae*
	I56		*Saccharomyces cerevisiae*
	I61		*Saccharomyces cerevisiae*
	I68		*Saccharomyces cerevisiae*
	CL1		*Saccharomyces cerevisiae*
	CL7		*Saccharomyces cerevisiae*
	CL8		*Saccharomyces cerevisiae*
	CL10		*Saccharomyces cerevisiae*
	CL14		*Saccharomyces cerevisiae*
	CL16		*Saccharomyces cerevisiae*
	CL19		*Saccharomyces cerevisiae*
	CL26		*Saccharomyces cerevisiae*
	CL27		*Saccharomyces cerevisiae*

	CL34		*Saccharomyces cerevisiae*
	E11		*Saccharomyces cerevisiae*
	E12		*Saccharomyces cerevisiae*
	E19		*Saccharomyces cerevisiae*
	E58		*Saccharomyces cerevisiae*
	E63		*Saccharomyces cerevisiae*
	E65		*Saccharomyces cerevisiae*
	E67		*Saccharomyces cerevisiae*
	E68		*Saccharomyces cerevisiae*
	E74		*Saccharomyces cerevisiae*
	E77		*Saccharomyces cerevisiae*
	I15	*Candida*	*Candida apicola*
	SH29		*Saccharomyces cerevisiae*
	AC29		*Saccharomyces cerevisiae*
	BH33		*Saccharomyces cerevisiae*
	SC37		*Saccharomyces cerevisiae*
	AH39		*Saccharomyces cerevisiae*
Bei Jing	LH21	*Saccharomyces*	*Saccharomyces cerevisiae*
	BH8		*Saccharomyces cerevisiae*
	LH39		*Saccharomyces cerevisiae*
	L59		*Saccharomyces cerevisiae*
	LB1		*Saccharomyces cerevisiae*

Xin Jiang	FSF19	*Candida*	*Candida glabrata*
	BG3		*Candida glabrata*
	XJA1	*Saccharomyces*	*Saccharomyces cerevisiae*
	XJA2		*Saccharomyces cerevisiae*
	XJA8		*Saccharomyces cerevisiae*
Yun Nan	B38	*Saccharomyces*	*Saccharomyces cerevisiae*
	B42		*Saccharomyces cerevisiae*
	B43		*Saccharomyces cerevisiae*
	B46		*Saccharomyces cerevisiae*
	B47		*Saccharomyces cerevisiae*
	B49		*Saccharomyces cerevisiae*
	B50		*Saccharomyces cerevisiae*
Shan Xi	SXC6	*Saccharomyces*	*Saccharomyces cerevisiae*
	SXC8		*Saccharomyces cerevisiae*
	SXC9		*Saccharomyces cerevisiae*
	SXC13		*Saccharomyces cerevisiae*
	SXC11	*Hanseniaspora*	*Hanseniaspora uvarum*
Ning Xia	D20	*Saccharomyces*	*Saccharomyces cerevisiae*
	D22		*Saccharomyces cerevisiae*
	D27		*Saccharomyces cerevisiae*
	D28		*Saccharomyces cerevisiae*
	D30		*Saccharomyces cerevisiae*
	D35		*Saccharomyces cerevisiae*

	D39		*Saccharomyces cerevisiae*
	D41		*Saccharomyces cerevisiae*
	LN21		*Saccharomyces cerevisiae*
	NC1		*Saccharomyces cerevisiae*
	NC3		*Saccharomyces cerevisiae*
	NW9		*Saccharomyces cerevisiae*
	NW12		*Saccharomyces cerevisiae*
	D1		*Candida xestobii*
	D16		*Candida globrata*
	D18		*Candida globrata*
	D40		*Candida globrata*
	LN9	*Candida*	*Candida globrata*
	LN10		*Candida globrata*
	LN12		*Candida tropicalis*
	LN13		*Candida tropicalis*
	LN22		*Candida tropicalis*
	H58		*Saccharomyces cerevisiae*
	H63		*Saccharomyces cerevisiae*
	H66		*Saccharomyces cerevisiae*
Shan Dong	H68	*Saccharomyces*	*Saccharomyces cerevisiae*
	H70		*Saccharomyces cerevisiae*
	YT13		*Saccharomyces cerevisiae*
	YT28		*Saccharomyces cerevisiae*

	YT2		*Candida globrata*
	YT3		*Candida globrata*
	YT4	*Candida*	*Candida globrata*
	YT10		*Candida globrata*
	YT25		*Candida globrata*
	GS5		*Saccharomyces cerevisiae*
	GS9		*Saccharomyces cerevisiae*
	GS29		*Saccharomyces cerevisiae*
	GS32	*Saccharomyces*	*Saccharomyces cerevisiae*
	GS33		*Saccharomyces cerevisiae*
	GS39		*Saccharomyces cerevisiae*
	GS40		*Saccharomyces cerevisiae*
	GS10		*Candida globrata*
Gan Su	GS11		*Candida globrata*
	GS12		*Candida globrata*
	GS13		*Candida globrata*
	GS14		*Candida globrata*
	GS15	*Candida*	*Candida globrata*
	GS16		*Candida globrata*
	GS18		*Candida globrata*
	GS20		*Candida globrata*
	GS22		*Candida globrata*
	GS27		*Candida globrata*

GS30		*Candida globrata*
GS31		*Candida globrata*
GS8	*Hanseniaspora*	*Hanseniaspora uvarum*

Table S2 Mulberry wine fermentation treatment group

Group	Fermentation	Strain	
		non-Saccharomyces cerevisiae	*Saccharomyces cerevisiae*
Control	Single	-	AMR-1
Treatment 1	Single	-	GS32
Treatment 2	Single	D1	-
Treatment 3	Subsequent	GS13	AMR-1
Treatment 4	Subsequent	D1	AMR-1
Treatment 5	Subsequent	GS13	GS32
Treatment 6	Subsequent	D1	GS32

Table S4 CIELab color parameters of mulberry juice and mulberry wine

Index	Mulberry juice	Mulberry wine						
		AMR-1 (CK)	GS32	D1	AMR-1+GS13	AMR-A+D1	GS32+GS13	GS32+D1
L^* value	46.40±0.91^{a}	35.01±3.12^{b}	35.80±0.60^{b}	31.63±1.47^{b}	34.55±1.68^{b}	34.95±3.22^{b}	20.77±3.24^{c}	16.31±3.90^{c}
a^* value	50.84±0.82^{a}	50.27±1.16^{a}	39.32±2.25^{b}	49.51±2.67^{a}	50.74±1.33^{a}	47.54±2.61^{a}	47.31±2.23^{a}	35.53±2.10^{b}
b^* value	17.93±1.02^{de}	21.64±0.13^{bd}	16.78±0.95^{e}	26.51±2.03^{a}	26.03±0.79^{ab}	24.90±2.99^{abc}	21.55±2.79^{cd}	7.69±2.73^{f}
C^* value	53.91±1.11^{ab}	53.82±2.16^{ab}	40.28±2.77^{c}	57.53±1.59^{a}	55.97±2.53^{ab}	51.24±1.66^{b}	54.50±2.37^{ab}	37.42±3.46^{c}
H^* value	0.34±0.01^{b}	0.41±0.02^{a}	0.34±0.05^{b}	0.47±0.03^{a}	0.44±0.06^{a}	0.46±0.03^{a}	0.41±0.06^{a}	0.19±0.03^{c}

Note: Different letter superscripts in the same row of the table indicate that there are significant differences in this index, $p<0.05$; CK stands for contrast.

Table S3 The results of indexes of mulberry juice and mulberry wine

	Mulberry juice	Mulberry wine						
		AMR-1 (CK)	GS32	D1	AMR-1+GS13	AMR-1+D1	GS32+GS13	GS32+D1
Brix	21.20±0.20^{a}	7.63±0.23^{de}	7.17±0.21^{e}	8.87±0.31^{b}	7.97±0.31^{cd}	8.03±0.15^{cd}	8.27±0.61^{bd}	8.37±0.65^{bc}
pH	3.67±0.04^{d}	3.84±0.03^{ab}	3.90±0.01^{a}	3.72±0.02^{cd}	3.74±0.06^{c}	3.75±0.03^{c}	3.82±0.04^{b}	3.75±0.04^{c}
Alcohol concentration (%)	N.D.	10.93±0.21^{a}	10.53±0.15^{b}	10.40±0.10^{bc}	10.47±0.25^{bc}	10.37±0.10^{bc}	10.33±0.21^{bc}	10.20±0.10^{c}
Total acid (g/L)	7.67±0.61^{d}	9.55±1.11^{c}	8.11±0.97^{d}	12.66±0.41^{a}	10.71±0.43^{bc}	10.80±0.84^{b}	12.16±0.38^{a}	11.60±0.53^{ab}
Total sugar (g/L)	N.D.	3.60±0.36^{c}	3.50±0.17^{c}	11.13±1.19^{a}	4.43±0.75^{c}	4.43±0.40^{c}	6.70±0.46^{b}	7.27±0.62^{b}
Volatile acid (g/L)	N.D.	4.36±0.67^{bd}	3.51±0.33^{e}	5.68±0.51^{a}	4.50±0.52^{bc}	4.27±0.27^{cde}	5.17±0.43^{ab}	5.83±0.50^{a}
Pyruvic acid (mg/L)	N.D.	68.23±5.56^{e}	71.02±7.94^{e}	128.97±7.45^{bc}	186.68±16.29^{a}	86.41±13.20^{de}	114.12 ±16.50^{cd}	144.14±12.69^{b}
Malic acid (g/L)	5.30±0.66^{a}	2.87±0.32^{b}	3.00±0.17^{b}	1.97±0.21^{c}	1.63±0.45^{c}	1.70±0.10^{c}	2.13±0.35^{c}	2.00±0.30^{c}
Total phenols (mg/L)	1375.80±56.97^{bc}	1590.72±244.85^{b}	1921.50±102.21^{a}	1451.23 ±29.91^{bc}	1381.35 ±100.18^{bc}	1518.26 ±285.04^{b}	1406.91 ±214.44^{bc}	1179.73 ±64.56^{c}

Note: "N.D." indicates that it was not detected. Different letter superscripts in the same row of the table indicate that there are significant differences in this index, $p<0.05$; CK stands for contrast.

Table S5 Summary of CIELab color parameter in mulberry wine during aging

Storage temperature	Storage time	CIELab index	Mulberry wine						
			AMR-1 (CK)	GS32	D1	AMR-1+GS13	AMR-A+D1	GS32+GS13	GS32+D1
Initial value	Initial value	L^* value	35.01±3.12^{b}	35.80±0.60^{b}	31.63±1.47^{b}	34.55±1.68^{b}	34.95±3.22^{b}	20.77±3.24^{c}	16.31±3.90^{c}
		a^* value	50.27±1.16^{a}	39.32±2.25^{b}	49.51±2.67^{a}	50.74±1.33^{a}	47.54±2.61^{a}	47.31±2.23^{a}	35.53±2.10^{b}
		b^* value	21.64±0.13^{bd}	16.78±0.95^{e}	26.51±2.03^{a}	26.03±0.79^{ab}	24.90±2.99^{abc}	21.55±2.79^{cd}	7.69±2.73^{f}
		C^* value	53.82±2.16^{ab}	40.28±2.77^{c}	57.53±1.59^{a}	55.97±2.53^{ab}	51.24±1.66^{b}	54.50±2.37^{ab}	37.42±3.46^{c}
		H^* value	0.41±0.02^{a}	0.34±0.05^{b}	0.47±0.03^{a}	0.44±0.06^{a}	0.46±0.03^{a}	0.41±0.06^{a}	0.19±0.03^{c}
17°C	After 1 week	L^* value	36.87±0.38^{a}	37.54±2.21^{a}	35.89±3.65^{a}	36.91±2.67^{a}	35.46±1.48^{a}	36.37±4.68^{a}	36.78±4.20^{a}
		a^* value	58.12±1.48^{a}	55.49±1.17^{b}	58.86±0.54^{a}	58.05±0.94^{a}	58.07±0.46^{a}	58.43±0.40^{a}	57.72±1.32^{a}
		b^* value	32.92±4.07^{ab}	27.50±2.08^{b}	38.24±3.44^{a}	36.11±0.89^{a}	37.37±0.65^{a}	37.29±4.95^{a}	34.63±1.67^{a}
		C^* value	66.85±3.28^{a}	61.95±1.79^{b}	70.26±1.76^{a}	68.37±1.18^{a}	69.06±0.71^{a}	69.44±2.60^{a}	67.34±0.68^{a}
		H^* value	0.51±0.04^{ab}	0.46±0.03^{b}	0.58±0.04^{a}	0.56±0.01^{a}	0.57±0.00^{a}	0.57±0.06^{a}	0.54±0.03^{a}
	After 3 weeks	L^* value	39.22±2.58^{a}	39.04±1.92^{a}	36.63±3.13^{a}	37.97±1.45^{a}	38.05±0.46^{a}	37.77±4.98^{a}	37.94±3.41^{a}
		a^* value	57.44±1.17^{ab}	55.77±1.62^{b}	59.23±0.30^{a}	58.98±0.43^{a}	58.97±0.06^{a}	58.63±0.42^{a}	58.22±0.90^{a}
		b^* value	31.18±4.54^{bc}	28.60±3.40^{c}	38.37±2.99^{a}	37.06±0.64^{ab}	36.66±0.74^{ab}	36.74±5.36^{ab}	34.80±1.27^{abc}
		C^* value	65.44±3.20^{bc}	62.72±2.99^{c}	70.66±1.43^{a}	69.66±0.70^{ab}	69.44±0.41^{ab}	69.34±2.95^{ab}	67.85±0.19^{ab}
		H^* value	0.49±0.05^{ab}	0.47±0.04^{b}	0.57±0.04^{a}	0.56±0.00^{a}	0.56±0.01^{a}	0.56±0.06^{a}	0.54±0.02^{ab}
	After 5 weeks	L^* value	37.10±1.11^{a}	38.92±1.21^{a}	36.85±3.42^{a}	38.74±0.66^{a}	37.79±1.68^{a}	36.83±4.91^{a}	38.46±3.50^{a}
		a^* value	57.42±1.16^{b}	55.45±1.34^{c}	59.17±0.19^{a}	58.96±0.24^{ab}	58.85±0.27^{ab}	58.63±0.77^{ab}	58.39±0.65^{ab}
		b^* value	33.99±3.11^{ab}	29.93±1.84^{b}	38.95±3.59^{a}	37.73±1.01^{a}	38.24±0.27^{a}	36.68±4.40^{a}	35.82±1.70^{a}
		C^* value	66.76±2.51^{b}	63.02±2.03^{c}	70.90±1.86^{a}	70.00±0.75^{ab}	70.18±0.26^{ab}	70.35±2.22^{ab}	68.52±0.45^{ab}

		H* value	0.53±0.03^{ab}	0.49±0.02^{b}	0.58±0.04^{a}	0.57±0.01^{a}	0.58±0.00^{a}	0.58±0.05^{a}	0.55±0.03^{ab}
		L* value	35.83±0.79^{a}	37.84±1.42^{a}	34.47±3.93^{a}	37.39±1.92^{a}	37.81±1.42^{a}	36.47±0.07^{a}	37.31±3.63^{a}
		a* value	56.78±0.97^{ab}	54.33±1.01^{bc}	57.35±0.67^{a}	56.97±0.47^{ab}	57.40±0.99^{a}	56.63±1.83^{ac}	53.73±2.62^{c}
	After 1 week	b* value	31.62±3.36^{b}	26.37±1.61^{c}	36.57±2.39^{a}	33.95±1.12^{ab}	34.15±0.82^{a}	36.06±0.37^{a}	30.16±1.08^{bc}
		C* value	65.04±2.49^{ab}	60.41±1.53^{c}	68.06±0.83^{a}	66.33±0.69^{a}	66.79±1.27^{a}	67.15±1.37^{a}	67.95±2.46^{bc}
		H* value	0.51±0.04^{b}	0.45±0.02^{c}	0.57±0.03^{a}	0.54±0.01^{ab}	0.54±0.00^{ab}	0.57±0.02^{a}	0.51±0.02^{b}
		L* value	36.28±0.88^{a}	38.02±1.85^{a}	37.50±2.55^{a}	38.02±1.24^{a}	36.66±1.50^{a}	39.70±0.85^{a}	37.46±3.41^{a}
		a* value	36.28±0.88^{a}	54.78±1.51^{b}	58.87±0.58^{a}	58.46±0.10^{a}	58.49±0.23^{a}	58.04±0.79^{a}	57.89±0.73^{a}
22°C	After 3 weeks	b* value	33.99±2.88^{ab}	29.73±2.60^{b}	37.34±2.30^{a}	37.49±0.46^{a}	38.33±0.46^{a}	37.70±3.77^{a}	35.55±1.35^{a}
		C* value	66.70±2.34^{a}	62.35±2.55^{b}	69.74±1.33^{a}	69.45±0.31^{a}	69.93±0.18^{a}	69.30±1.56^{a}	68.43±0.17^{a}
		H* value	0.53±0.03^{ab}	0.50±0.03^{b}	0.56±0.03^{a}	0.57±0.01^{a}	0.58±0.01^{a}	0.57±0.05^{a}	0.55±0.02^{ab}
		L* value	34.98±1.01^{a}	35.94±1.84^{a}	34.98±2.94^{a}	37.05±1.00^{a}	35.20±1.51^{a}	38.36±1.70^{a}	37.23±2.79^{a}
		a* value	56.67±1.18^{a}	54.85±0.94^{b}	58.24±0.49^{a}	58.06±0.17^{a}	57.96±0.31^{a}	57.28±1.45^{a}	57.66±0.48^{a}
	After 5 weeks	b* value	36.08±2.88^{ab}	32.42±2.14^{b}	39.95±2.45^{a}	39.50±1.22^{a}	40.04±0.28^{a}	38.58±2.01^{a}	36.82±1.46^{a}
		C* value	67.21±2.43^{b}	63.73±1.88^{c}	70.66±1.21^{a}	70.23±0.82^{a}	70.45±0.37^{a}	69.10±0.38^{ab}	57.66±0.70^{ab}
		H* value	0.57±0.03^{ab}	0.53±0.02^{b}	0.60±0.03^{a}	0.60±0.01^{a}	0.60±0.00^{a}	0.59±0.04^{a}	0.57±0.02^{ab}
		L* value	38.63±1.10^{a}	38.56±3.59^{a}	31.50±3.42^{b}	35.08±1.49^{ab}	34.70±3.24^{ab}	31.88±3.79^{b}	33.82±1.86^{ab}
		a* value	56.30±0.82^{a}	53.40±2.87^{b}	54.48±0.65^{ab}	54.23±0.65^{ab}	54.52±0.57^{ab}	54.74±0.66^{ab}	53.09±0.73^{b}
	After 1 week	b* value	32.44±1.61^{ab}	29.43±3.77^{b}	35.21±2.43^{a}	32.76±0.75^{ab}	33.85±2.50^{ab}	35.91±2.12^{a}	31.72±1.77^{ab}
37°C		C* value	64.99±1.38^{a}	61.00±4.31^{a}	64.89±1.75^{a}	62.15±2.55^{a}	64.20±1.75^{a}	65.49±1.26^{a}	61.86±1.49^{a}
		H* value	0.52±0.02^{abc}	0.50±0.03^{c}	0.57±0.03^{ab}	0.52±0.04^{bc}	0.55±0.03^{abc}	0.58±0.03^{a}	0.54±0.02^{abc}
	After 2 weeks	L* value	32.75±2.42^{a}	33.89±1.42^{a}	31.25±2.38^{a}	34.00±1.91^{a}	32.44±2.65^{a}	32.37±4.54^{a}	33.76±2.71^{a}
		a* value	54.53±0.51^{ab}	53.42±0.73^{b}	54.89±0.25^{ab}	55.80±0.25^{a}	55.62±1.49^{a}	54.97±0.77^{ab}	55.03±0.55^{ab}

	b* value	35.80±1.71^{bc}	33.55±1.20^{c}	37.81±1.73^{ab}	38.79±0.68^{ab}	39.19±2.31^{a}	37.41±1.43^{ab}	36.24±0.86^{abc}
	C* value	65.25±1.11^{bc}	63.09±1.17^{c}	66.66±1.18^{ab}	67.96±0.58^{ab}	68.05±2.52^{a}	66.51±0.85^{ab}	65.89±0.70^{ab}
	H* value	0.58±0.02^{ab}	0.56±0.01^{b}	0.60±0.02^{a}	0.61±0.01^{a}	0.61±0.02^{a}	0.60±0.02^{a}	0.58±0.01^{ab}
After 3 weeks	L* value	39.19±0.83^{a}	36.21±3.34^{a}	34.37±2.87^{a}	36.87±1.25^{a}	36.10±3.66^{a}	34.68±3.87^{a}	40.07±2.38^{a}
	a* value	53.67±1.38^{b}	53.35±1.46^{b}	55.03±0.83^{ab}	56.07±0.31^{a}	56.11±0.91^{a}	54.25±0.83^{ab}	54.54±0.56^{ab}
	b* value	34.34±1.49^{c}	35.68±1.55^{bc}	37.77±2.49^{ac}	39.41±1.09^{a}	39.08±0..86^{ab}	36.59±0.79^{abc}	34.43±2.18^{c}
	C* value	63.55±1.96^{c}	64.18±2.02^{bc}	66.77±2.01^{ab}	68.54±0.72^{a}	68.38±0.83^{a}	65.44±0.25^{abc}	64.53±1.46^{bc}
	H* value	0.57±0.01^{bc}	0.59±0.01^{abc}	0.60±0.03^{ab}	0.61±0.01^{a}	0.61±0.01^{ab}	0.59±0.02^{abc}	0.56±0.03^{c}
After 4 weeks	L* value	36.39±1.92^{a}	35.30±1.56^{a}	35.15±4.34^{a}	37.57±1.91^{a}	38.09±3.03^{a}	36.58±3.14^{a}	35.21±1.26^{a}
	a* value	53.69±1.53^{a}	50.42±2.10^{b}	53.49±0.66^{a}	53.04±1.23^{ab}	53.59±1.37^{a}	55.61±1.63^{a}	54.56±0.48^{a}
	b* value	37.01±1.74^{ab}	32.35±2.71^{b}	36.02±2.92^{ab}	40.78±3.68^{a}	36.28±1.94^{ab}	35.54±0.47^{b}	36.60±1.33^{ab}
	C* value	65.22±2.12^{ab}	59.92±3.20^{c}	64.52±2.17^{abc}	68.99±3.51^{a}	64.72±2.17^{abc}	63.85±1.28^{bc}	65.71±1.13^{ab}
	H* value	0.60±0.01^{ab}	0.57±0.02^{b}	0.59±0.03^{ab}	0.63±0.03^{a}	0.59±0.02^{ab}	0.59±0.01^{ab}	0.59±0.01^{ab}
After 5 weeks	L* value	32.73±1.23^{a}	33.43±0.41^{a}	33.55±3.52^{a}	34.68±2.52^{a}	33.52±2.49^{a}	35.00±2.09^{a}	36.24±3.93^{a}
	a* value	52.55±1.07^{ab}	52.04±1.05^{b}	53.90±0.49^{ab}	53.94±0.42^{a}	53.92±0.92^{a}	52.87±1.28^{ab}	53.08±0.38^{ab}
	b* value	38.27±1.47^{abc}	37.82±0.83^{abc}	39.08±2.08^{ab}	40.24±0.81^{a}	39.33±0.80^{ab}	36.74±1.53^{bc}	35.72±1.20^{c}
	C* value	65.01±1.68^{ab}	64.33±1.32^{ab}	66.59±1.60^{ab}	67.29±0.80^{a}	66.74±1.19^{ab}	64.39±1.83^{ab}	63.98±0.98^{b}
	H* value	0.63±0.01^{ab}	0.63±0.00^{ab}	0.63±0.02^{ab}	0.64±0.01^{a}	0.63±0.00^{ab}	0.61±0.01^{bc}	0.59±0.01^{c}

Note: Different letter superscripts in the same row of the table indicate that there are significant differences in this index, $p<0.05$; CK stands for contrast.

Table S6 Content of volatile components in mulberry wine

Volatile components	Mulberry wine						
	AMR-1 (CK)	GS32	D1	AMR-1+GS13	AMR-1+D1	GS32+GS13	GS32+D1
Esters（μg/L）							
Methyl acetate	166.76±8.97[a]	194.03±9.60[a]	122.50±7.88[c]	131.53±11.87[c]	134.83±7.61[c]	173.11±4.09[b]	208.27±7.53[a]
Ethyl acetate	1023.48±107.99[a]	644.06±58.08[c]	810.22±86.67[b]	1037.83±123.07[a]	685.73±46.57[bc]	726.28±87.22[bc]	738.06±105.70[bc]
Ethyl propionate	5.56±1.25[bc]	6.74±0.38[b]	6.02±1.96[bc]	4.75±0.32[c]	6.10±0.89[bc]	11.90±0.70[a]	5.35±0.84[bc]
Ethyl butyrate	---	1047.65±91.66[a]	---	397.44±5.96[b]	---	384.83±18.96[b]	317.16±11.30[b]
Diethyl succinate	---	89.97±9.37[b]	374.78±20.68[a]	45.18±1.98[c]	---	51.02±4.55[c]	44.22±2.60[c]
Ethyl nonanoate	1601.12±82.71[a]	---	---	---	446.58±40.34[b]	---	---
Ethyl stearate	4.60±1.06[b]	---	69.31±1.42[a]	---	5.61±0.63[b]	---	---
Ethyl undecanoate	23.43±1.41[ab]	---	26.41±2.46[a]	---	20.54±2.51[b]	---	---
Isoamyl acetate	8.65±0.55[d]	---	7.57±0.66[d]	208.14±25.40[c]	7.03±0.59[d]	314.48±29.90[a]	250.28±12.91[b]
Ethyl valerate	---	68.98±0.95[a]	---	---	---	---	---
Methyl-9-octadecenoate	191.78±4.18[d]	3.25±0.30[e]	231.45±41.18[b]	2.13±0.29[e]	294.79±26.17[a]	2.10±0.08[e]	3.79±1.87[e]
Ethyl myristate	71.88±6.85[a]	5.20±0.45[d]	18.94±1.58[c]	4.65±0.31[d]	45.07±1.68[b]	5.48±0.63[d]	5.27±1.49[d]
Ethyl palmitate	428.81±44.30[a]	307.54±32.64[b]	398.26±30.28[a]	187.25±46.31[c]	390.32±70.22[a]	238.45±44.55[bc]	200.55±20.27[c]
Ethyl heptanoate	18.74±1.21[b]	25.93±2.88[a]	18.78±1.50[b]	18.05±1.06[b]	13.55±3.84[c]	16.53±1.32[bc]	19.41±0.99[b]
Methyl caprylate	3.61±0.44[c]	74.75±9.06[b]	10.28±2.49[c]	119.73±39.84[a]	6.20±1.19[c]	119.99±16.25[a]	104.22±13.43[ab]
Methyl benzoate	286.99±14.75[a]	3.60±0.29[d]	186.10±31.40[c]	3.96±1.28[d]	228.27±20.52[b]	3.74±0.09[d]	3.32±0.07[d]
Ethyl heptadecanoate	135.37±14.47[a]	---	125.89±15.42[a]	---	134.94±6.61[a]	---	---
Ethyl caprylate	29.80±1.51[a]	5.21±0.44[b]	6.91±0.47[b]	24.25±4.79[a]	6.93±1.08[b]	5.76±0.61[b]	3.51±1.22[b]
Ethyl benzoate	60.70±7.92[a]	21.80±1.77[c]	50.59±2.78[b]	12.62±2.52[d]	45.21±3.48[b]	13.09±1.07[d]	12.55±1.35[d]
Methyl salicylate	12.79±1.91[a]	2.16±0.13[b]	12.92±0.83[a]	---	12.49±2.13[a]	---	1.15±0.21[b]

Ethyl phenylacetate	2.99±0.82^{c}	18.94±4.12^{a}	3.46±0.64^{c}	22.37±3.15^{a}	3.38±0.91^{c}	13.46±1.87^{b}	---
Phenethyl acetate	---	35.27±9.46^{a}	1.82±0.35^{c}	8.90±0.09^{b}	---	8.45±0.27^{b}	10.83±0.84^{b}
Ethyl caprate	38.40±4.74^{a}	---	14.93±2.48^{b}	1.35±0.32^{c}	11.22±1.67^{b}	1.33±0.11^{c}	1.88±0.98^{c}
Monoethyl succinate	19.69±1.59^{a}	1.84±0.35^{c}	14.41±0.64^{b}	---	12.65±1.70^{b}	1.68±0.63^{c}	---
Ethyl laurate	205.73±15.52^{a}	1.15±0.13^{c}	63.28±5.45^{b}	---	60.53±9.48^{b}	1.68±0.63^{c}	---
Diethyl phthalate	4.51±1.61^{c}	2.35±0.73^{cd}	1.49±0.21^{d}	10.98±2.31^{b}	1.41±0.30^{d}	14.99±1.93^{a}	13.88±1.87^{a}
Aldehydes and ketones（μg/L）							
Acetaldehyde	106.76±2.97^{b}	134.03±5.60^{a}	62.50±6.88^{c}	71.53±8.87^{c}	74.83±7.61^{c}	113.11±4.09^{b}	148.27±7.53^{a}
3-Hydroxybutyraldehyde	---	---	33.08±1.28^{a}	---	---	---	---
4-Pentenal	202.99±6.42^{a}	122.16±6.36^{b}	57.24±6.95^{c}	51.23±5.64^{cd}	54.65±6.92^{cd}	46.02±6.21^{d}	47.47±5.08^{cd}
2-Nonenal	39.20±1.34^{a}	34.97±3.05^{ab}	31.59±2.52^{b}	32.37±0.95^{b}	31.22±3.23^{bc}	27.06±2.43^{cd}	25.31±3.09^{d}
Decanal	---	39.81±3.26^{a}	---	6.24±1.01^{b}	---	6.40±0.70^{b}	7.41±0.33^{b}
2,3-Pentanedione	7.36±1.16^{b}	17.83±2.15^{a}	4.06±1.53^{c}	7.76±1.52^{b}	4.93±1.33^{c}	8.43±1.48^{b}	5.16±0.94^{c}
beta-Damascenone	35.03±1.94^{a}	3.21±0.35^{c}	10.17±0.78^{b}	1.62±0.35^{c}	12.15±3.57^{b}	1.17±0.04^{c}	2.86±0.65^{c}
2-Octanone	---	385.22±44.30^{a}	2.03±1.25^{c}	47.76±2.61^{b}	1.15±0.11^{c}	47.61±3.77^{b}	54.48±3.04^{b}
Acetophenone	4.86±1.08^{cd}	102.21±3.82^{a}	3.99±0.50^{d}	7.83±0.80^{bc}	5.01±0.08^{cd}	8.87±0.59^{b}	11.05±1.32^{b}
Terpenes（μg/L）							
Citronellol	78.52±7.96^{a}	15.47±0.87^{c}	48.37±4.02^{b}	8.45±0.77^{d}	45.38±2.11^{b}	5.09±1.54^{d}	5.46±0.43^{d}
Terpinenes	23.91±1.27^{a}	6.78±1.95^{b}	26.72±3.72^{a}	---	28.13±1.89^{a}	---	---
Terpinolene	---	304.01±17.58^{d}	---	357.22±8.60^{c}	---	504.54±43.19^{a}	442.99±20.68^{b}
Linalool	17.31±0.45^{b}	---	17.26±1.85^{b}	---	21.26±1.00^{a}	---	---
Citronellal	8.74±0.33^{c}	72.61±3.82^{a}	10.03±2.37^{c}	45.28±5.57^{b}	8.22±0.42^{c}	46.01±6.21^{b}	47.87±6.22^{b}
Terpinen-4-ol	16.36±0.80^{a}	1.72±0.44^{c}	14.87±2.62^{a}	1.73±0.48^{c}	11.36±0.11^{b}	1.20±0.17^{c}	---

Nerol	22.65±2.46[ab]	---	18.71±3.97[b]	1.41±0.28[c]	24.76±2.71[a]	---	---
Acid（μg/L）							
Pentylarsonic acid	---	19.34±1.69[ab]	---	15.48±1.84[b]	---	23.03±4.69[a]	16.77±1.85[b]
Benzoic acid	37.11±5.01[a]	11.26±4.77[c]	21.44±1.26[b]	3.02±0.58[d]	13.70±1.41[c]	2.20±0.19[d]	2.35±0.42[d]
Nonanoic acid	3.83±0.27[b]	15.48±4.32[a]	3.90±0.77[b]	12.71±2.14[a]	3.97±1.48[b]	14.32±1.40[a]	13.09±1.50[a]
3-Hydroxydodecanoic acid	9.75±0.58[b]	13.94±1.16[a]	2.56±0.49[c]	1.84±0.80[c]	2.01±0.47[c]	2.02±0.54[c]	1.71±0.55[c]
Nonanoic acid	19.66±1.19[a]	1.60±0.46[b]	4.95±1.07[b]	1.87±0.68[b]	---	1.18±0.10[b]	1.68±0.64[b]
Hexadecenoic acid	7.21±0.59[c]	2.27±0.97[d]	14.18±1.43[a]	1.75±0.72[d]	12.18±0.73[b]	1.19±0.08[d]	1.56±0.46[d]
Alcohols（μg/L）							
Propanol	139.95±11.68[a]	120.65±18.68[b]	15.22±2.88[e]	55.64±4.99[c]	20.77±3.07[e]	33.78±4.40[d]	42.25±4.28[cd]
Diisobutylcarbinol	145.92±16.27[a]	29.62±1.44[c]	44.60±1.35[b]	23.81±1.09[c]	1.76±0.19[d]	23.47±2.92[c]	23.59±1.93[c]
1-Butanol	5.32±0.36[c]	13.05±0.69[b]	2.50±0.51[c]	20.65±3.01[a]	4.61±0.87[c]	19.80±3.41[a]	14.04±2.47[b]
1-Nonanol	13.30±1.94[a]	1.40±0.08[d]	6.68±1.13[b]	1.11±0.10[d]	4.76±0.20[c]	---	2.09±0.60[d]
1-Dodecanol	18.86±2.91[c]	2.01±0.51[d]	53.81±1.12[a]	1.64±0.55[d]	30.05±1.71[b]	1.29±0.15[d]	1.83±0.34[d]
Other（μg/L）							
4-Ethylphenol	---	11.01±1.58[a]	1.53±0.47[c]	9.44±0.71[b]	1.49±0.53[c]	12.11±0.17[a]	10.82±0.46[a]
2,6-Di-tert-butylphenol	4.78±0.31[cd]	27.83±2.47[b]	6.66±1.96[c]	32.98±3.57[b]	1.56±0.54[d]	40.47±4.35[a]	34.76±1.64[ab]
Eugenol	3.08±0.39[c]	74.47±1.28[a]	3.35±0.38[c]	69.76±6.31[a]	4.93±0.83[c]	59.03±1.30[b]	51.95±3.11[b]
Coumarin	---	---	20.19±2.18[a]	---	---	---	---

Note: "--" indicates that it was not detected. Different letter superscripts in the same row of the table indicate that there are significant differences in this index, $p<0.05$; CK stands for contrast.

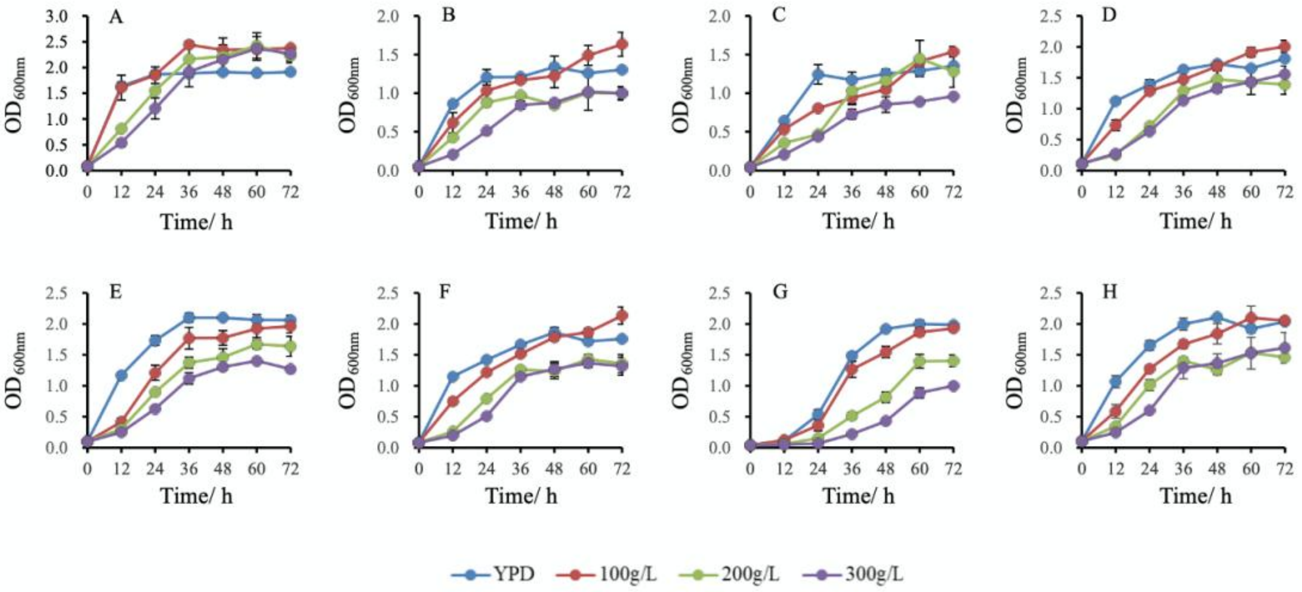

Figure S1 Growth curve of 8 yeast strains under different sugar concentrations. (A) GS32 strain; (B) SXC11 strain; (C) GS8 strain; (D) GS30 strain; (E) GS31 strain; (F) GS13 strain; (G) I15 strain; (H) D1 strain.

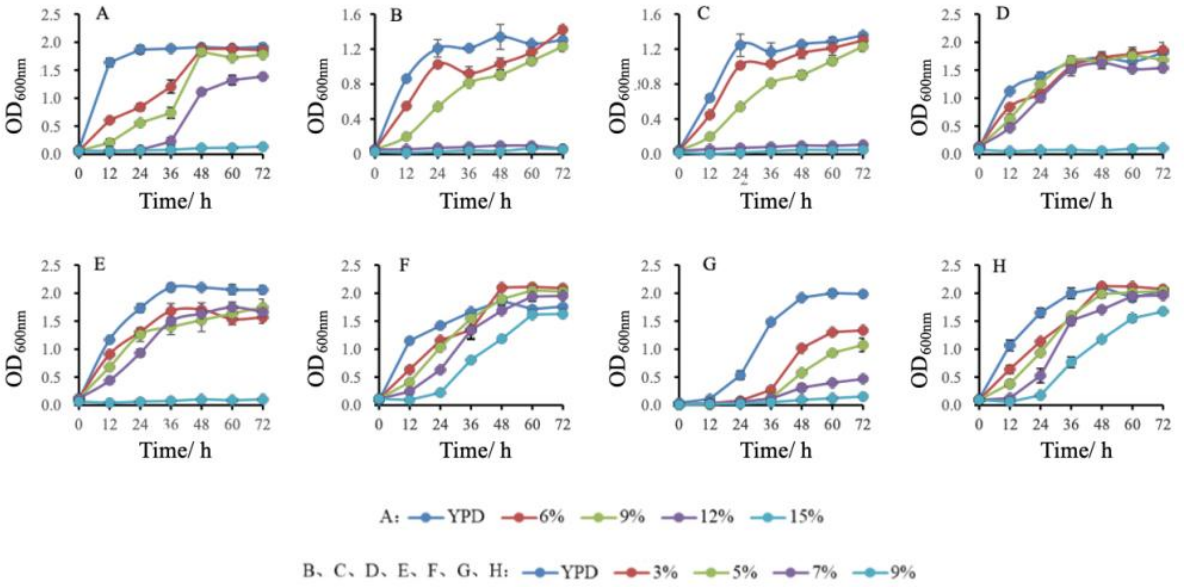

Figure S2 Growth curve of 8 yeast strains under different alcohol concentrations. (A) GS32 strain; (B) SXC11 strain; (C) GS8 strain; (D) GS30 strain; (E) GS31 strain; (F) GS13 strain; (G) I15 strain; (H) D1 strain.

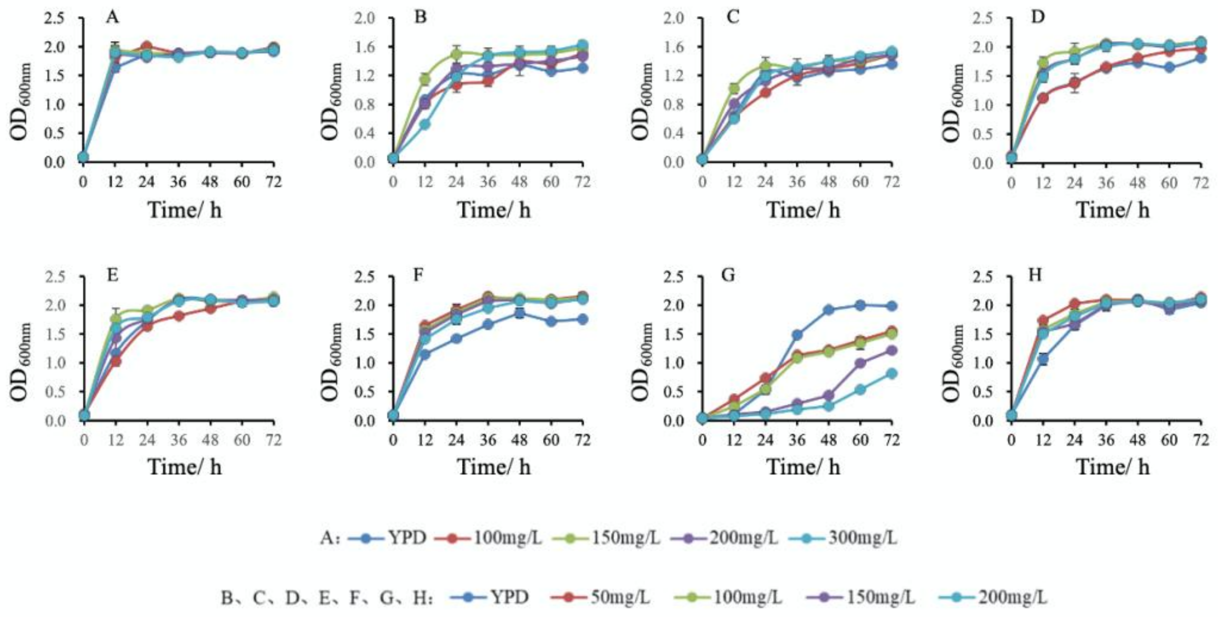

Figure S3 Growth curve of 8 yeast strains under different SO2 concentrations. (A) GS32 strain; (B) SXC11 strain; (C) GS8 strain; (D) GS30 strain; (E) GS31 strain; (F) GS13 strain; (G) I15 strain; (H) D1 strain.
